# Drive Through Robotics: Robotic Automation for Last Mile Distribution of Food and Essentials During Pandemics

**DOI:** 10.1109/ACCESS.2020.3007064

**Published:** 2020-07-06

**Authors:** Ajit Sharma, Philip Zanotti, Laxmi P. Musunur

**Affiliations:** 1 Mike Illitch School of BusinessWayne State University 2954 Detroit MI 48104 USA; 2 Fanuc America Rochester Hills MI 48309 USA

**Keywords:** Robotics, artificial intelligence, machine vision, robots, automation, digital simulation, digital twin, drive through, covid-19, pandemic, food security

## Abstract

The COVID-19 pandemic unraveled the weak points in the global supply chain for goods. Specifically, people all over the world, including those in the most advanced nations have had to go without medical supplies and personal protective equipment. Scarcity of essentials increases anxiety and uncertainty exacerbating unproductive behaviors like hoarding and price gouging. Left to market forces, such unfair practices are likely to aggravate hardships and increase the loss of lives. Thus, there is a critical need to ensure safe distribution of food and essential supplies to all citizens to sustain them through challenging times. To this end, we propose a simple, affordable and contact-less robotic system for preparing and dispensing food and survival-kits at community scale. The system has provisions to prevent hoarding and price gouging. Design, simulation, and, validation of the system has been completed to ensure readiness for real world implementation. This project is part of an open-source program and detailed designs are available upon request to entities interested in using it to serve their communities.

## I. Introduction

A critical aspect of responding to large scale medical emergencies like pandemics is a well functioning supply chain. In normal times supply chains need to balance the competing demands of flexibility and responsiveness. As the COVID-19 contagion has demonstrated, pandemics lead to shortages of goods and supplies. Shortages in turn trigger behavioral responses like hoarding and price gouging. Such tendencies surface at multiple levels in the supply chain. At the global level, countries may restrict the export of critical supplies like food [1] and medicines [2] to serve their own populations, or worse yet, threaten to weaponize supply chains on which they perceive a monopoly [3]. At the national level, states compete with the Federal Government and poach each other’s orders [4]. This tendency is repeated at the level of states [5], [6] and cities [7] which engage in a bidding war to acquire contested resources like Personal Protective Equipment (PPEs). In each of these cases, stronger and rule-bending entities may corner a disproportionate share, outbidding weaker ones. This may create scarcity amidst pockets of plenty, causing avoidable hardships and loss of lives.

A similar asymmetry in cornering resources is at play at the level of individual households. For instance, in the last mile of the food supply chain, the prevalence of food deserts is a well-known phenomenon [47]. A pandemic amplifies this disparity in access to essentials, at the fault-lines of poverty and the digital divide. Those with the means to pay the premiums, and, the requisite digital savvy, are able to search for and divert items in short supply to their doorsteps, using online channels.

The implication for those without the means is visiting stores to purchase food and essential supplies. This heightens their risk of exposure, as stores are likely hot spots for transmission of germs during pandemics. The majority of US citizens use large grocery stores as their primary way to acquire food, and, hence we have chosen them as the context for our study.

Studies of US household food acquisition behavior reveal that nearly all households acquire food at least once during the week; 87 percent visit large grocery stores and supermarkets, and 85 percent visit restaurants and other eating places at least once [8]. Due to quarantine restrictions during the COVID-19 pandemic, food acquisition of prepared meals reduced significantly, shifting the demand to grocery stores. This prompted the Government to classify groceries as an essential service and declare new guidelines which allow grocery workers to continue working even after exposure to COVID-19, as long as they remain asymptomatic [9]. Such policies are likely to further increase the risk of exposure during grocery store visits. We evaluate this risk of exposure to germs in three typical models for last mile fulfillment, by closely studying them in section II.

## II. Last Mile Fulfillment Models

### A. In-Store

**FIGURE 1.**
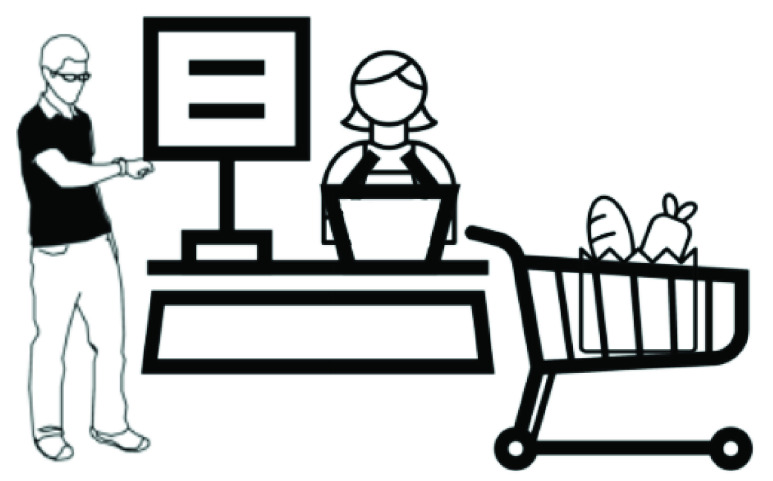
Traditional in-store shopping.

**FIGURE 2.**
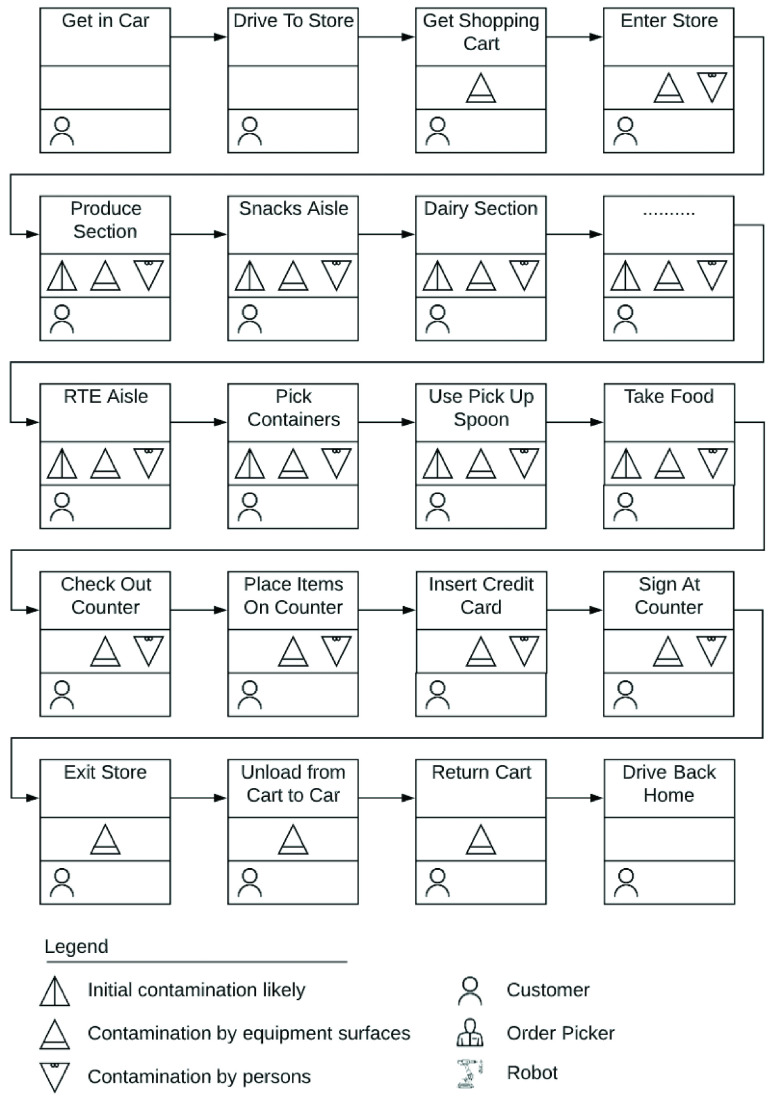
Process flow of grocery shopping trip.

In-store shopping in large grocery stores is the primary mode of food acquisition for most American households. Figure 1 represents traditional in-store shopping and Figure 2 lays out the process flow for a customer in a grocery store. In the process flow, potential points of transmission have been categorized into initial transmission, transmission by people or transmission through contact with a surface. We also classify each step into those executed by a customer, a grocery store worker or by an autonomous machine such as a robot.

### B. Curb-Side Pick-Up

**FIGURE 3.**
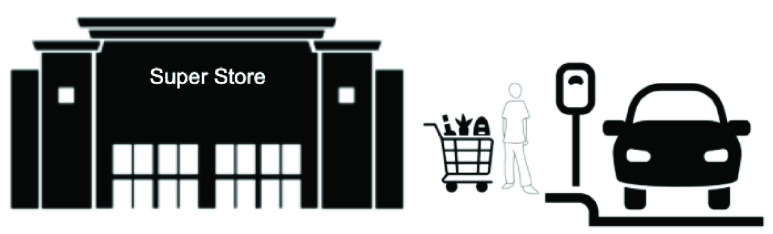
Curbside pick up.

**FIGURE 4.**
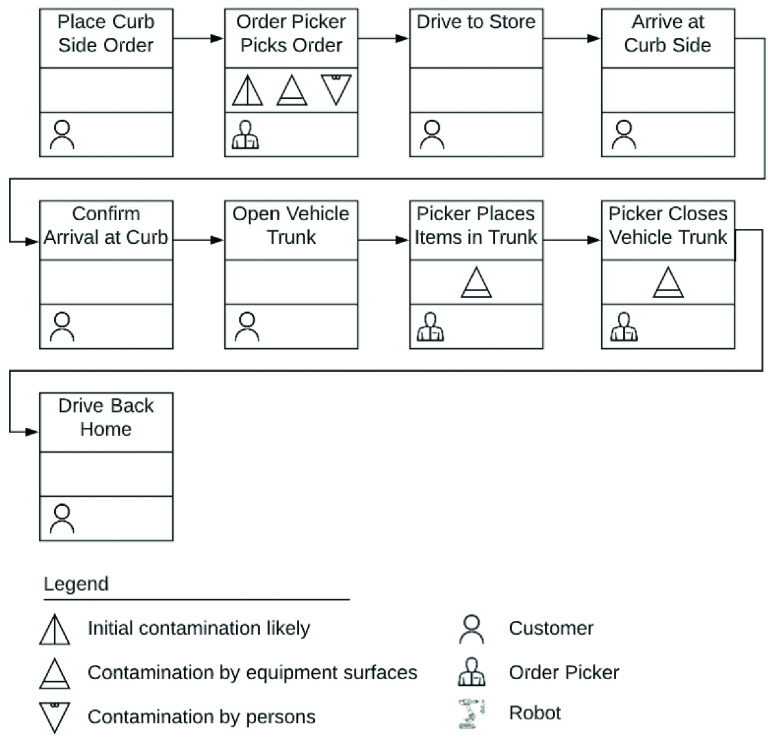
Process flow of curb side delivery order.

Curb side pickup is increasingly popular with customers [10]. In this model, the order is placed online and an in-store order-picker picks items for a customer. For pickup, the customer waits in her vehicle outside the store and a store worker brings the order to the curb to deliver it. This allows the customer to avoid entering the store and getting exposed to germs. A conceptual view of curb side pickup is illustrated in Figure 3. While curb-side pick-up reduces the chances of exposure substantially for a customer, employing human order-pickers adds to the direct operational costs of the store. It is estimated that for picking an order of $100, it takes around one hour which might cost a store an additional $20 [17]. Stores may pass on this cost to the customer in the form of additional fees, premium pricing, and, minimum order sizes. Tips may further increase the final out of pocket cost for customers. A process flow diagram of a curb side pick up order is presented in Figure 4.

### C. Home Delivery

Grocery sales through online platforms have traditionally been a very small part of the overall grocery retail market. However, it is the fastest growing segment [11]. Some of the companies in the US that have been trying to get a foothold in the online grocery market including AmazonFresh, FreshDirect, NetGrocer and Safeway. Third party delivery companies like Instacart and Shipt are helping establish home delivery as an important channel. The COVID-19 pandemic has further accelerated the shift towards online shopping. For instance, online sales for Target increased by 275% in the month of April, 2020 [12].

**FIGURE 5.**
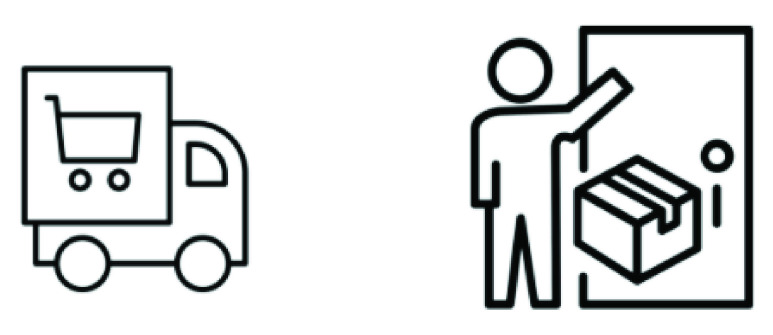
Home delivery.

**FIGURE 6.**
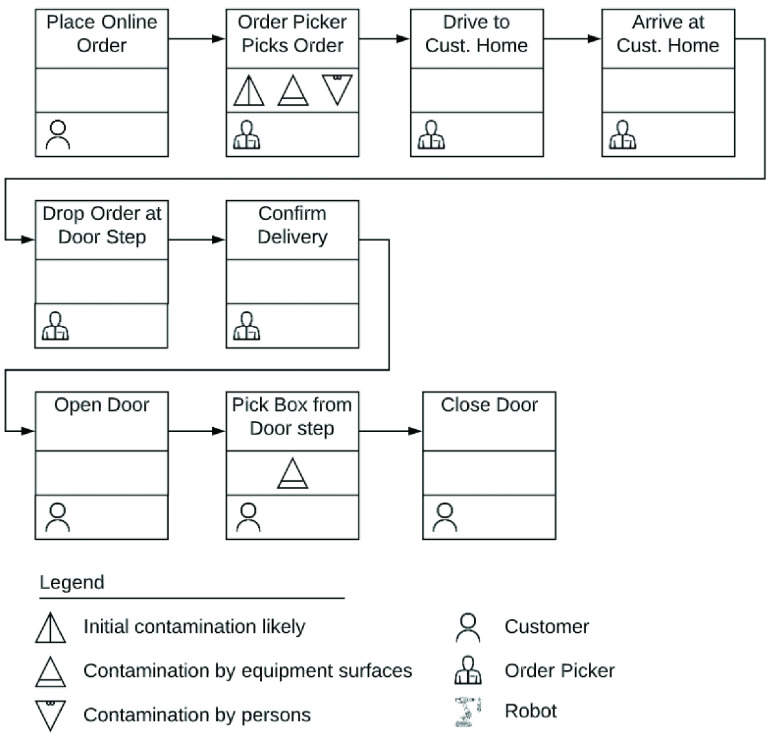
Process flow of home delivery order.

In Figure 5, we lay out a conceptual view, and, Figure 6 presents the flow of activities in the fulfillment of a home delivery order. While home delivery avoids exposure to stores, it comes with additional costs like delivery fees, premium pricing as well as tips. This might preclude the very populations facing the worst food security challenges.

## III. Last Mile Fulfilment Challenges

The COVID-19 has been shown to remain viable and infectious for hours in aerosols and for days on surfaces [13]. Seen in this light, Figures 2, 4, and, 6 clearly highlight the role of food packages and food handlers as potential carriers of disease. Contagion may be further amplified because the nature of grocery store work necessitates employees to work in close proximity. Additionally, evidence suggests that customers in grocery stores are unlikely to adhere to social distancing rules [14]. A survey of more than 5000 grocery and food workers found that over 85% of customers do not practice social distancing [15]. Hence, measures to reduce the risk of infection, including cleaning and disinfecting food, as well as social distancing guidelines, may fall short.

As long as last mile fulfillment involves human labor, and people being in close proximity, grocery stores may remain a vector for disease transmission. This may be reflected in the growing incidence of deaths of grocery store workers in the wake of COVID-19 [16].

Because groceries remain the main source of food, the Centers for Disease Control and Prevention (CDC) has issued temporary provisions to allow employees to continue to work even after being exposed to COVID-19, as long as they remain asymptomatic [19]. With the rise in death of grocery workers, unions have protested against such policy guidelines and there is a growing unwillingness to work [16], creating a shortage of workers. Shortage of workers, apart from generating long wait times in order fulfillment, also raises labor costs. For instance, order-pickers for online and curbside orders can account for up to 63% of operational costs for stores [17]. Every order, end to end, may take about one hour of labor to pick, consolidate, stage, and prepare for delivery. This can easily add an additional $20 to the order cost.

Because of these challenges during pandemics, it becomes imperative to have last mile fulfillment models that do not rely upon in-store customer visits or human employees. In section IV, we conduct an extensive review of the literature to find the existence of alternative models.

## IV. Literature Review

We conducted our literature search in two digital libraries: IEEE Xplore and Web of Science. We set out to find articles on the use of robots and automation in delivery of food. The search terms used and article counts are summarized in Table 1.

**TABLE 1:** Count of Articles on Use of Robots in Last Mile Delivery of Food

Database	Search Terms	Article Count
IEEE Xplore	Food AND	19
	Delivery AND	
	Robot*	
Web Of Science	Food AND	29
	Delivery AND	
	Robot*	

The search yielded a total of 48 articles. Five articles were duplicates which were removed from the result set. We also discarded 20 studies in domains unrelated to food, such as, robotic surgery, space exploration, nano-robots for drug delivery, use of robots in drug discovery, wireless sensor networks, and, MEMS (micro-electromechanical systems).

From the remaining 23 articles, we further eliminated those that were in the fields of agriculture and farming [20], food delivery using nano-particles [22], ingestible nano-robots for diagnosis [23], battlefield operations [24], and, feature descriptions of specific industrial robot models [21]. This yielded a reduced set of 18 articles. Finally, we also eliminated studies exploring the use of robots for feeding animals such as dogs [25] and fish [26]. Our final result set of studies on the use of robots in food delivery, consisted of 16 articles. Key attributes of each of these studies, including study context, environment of automation, purpose of automation, and, contribution of study are presented in Table 4.

**TABLE 2:** Tabulation of Articles on Dimensions of Technology and Nature of Contribution

CONTRIBUTED ARTEFACT	TECHNOLOGY					Grand Total
	Robot	Platform	IoT	Drone	AMV^1^	
Algorithm	4			2		6
Case Study	1					1
Design	1		2			3
Interpretation	1					1
Prototype	1					1
Review					1	1
Simulation	1					1
Software		1				1
Survey	1					1
Grand Total	10	1	2	2	1	16

**TABLE 3:** Tabulation of Articles on Dimensions of Technology and Application Context

APLN. CONTEXT	TECHNOLOGY					Grand Total
	Robot	Platform	IoT	Drone	AMV	
Restaurant	6		1			7
Last Mile Delivery			1	2	1	4
Hospital	3	1				4
Home	1					1
Grand Total	10	1	2	2	1	16

**TABLE 4:** Studies on Robotic Delivery of Food

Ref.#	Year	Title	Author	Environment	Context	Automation Artefact	Automation Artefact Detail	Automation Function	Study Contribution	Contribution Details
[27]	2018	Application of modified Asimov’s laws to the agent of home service robot using state, operator, and result (Soar)	Van Dang, C; et. al.	Indoors	Home	Robot	Home service robots	Complete Household Chores	Interpretation	Interpretation of Asimov’s Three Laws of Robotics
[28]	2015	OpenCRP Ecosystem Demonstration Platform	Oksa, P; et. al.	Indoors	Hospitals	Platform	Cloud based platform	Coordinate operation of a team of robots	Software	Open Source Cloud Based Platform
[29]	1999	A stereo vision system for position measurement and recognition in an autonomous robotic system for carrying food trays	F. Hasegawa; et. al.	Indoors	Hospitals	Robot	Food Tray carrying robot	Carry food trays in a restaraunt	Algorithm	Stereo Vision System Algorithm
[30]	1994	Experiments with a mobile robot operating in a cluttered unknown environment	Skewis, T; Lumelsky, V	Indoors	Hospitals	Robot	Mobile Hospital Robots	Delivery of food and medicines in a hospital	Algorithm	Motion Planning Algorithm for Navigating uncertain environments
[31]	2011	Mobile hospital robots cure numerous logistic needs	Bloss, R	Indoors	Hospitals	Robot	Mobile Hospital Robot	Complete numerous hospital tasks	Case Study	Case Study
[32]	2019	lot Intelligent Restaurant System Design	Deng, BQ; et. al.	Indoors	Restaraunt	IoT	IoT System	Automation and Intelligence in restaraunts	Design	System Design
[33]	2018	Food Delivery Automation in Restaurants Using Collaborative Robotics	A. Antony; P. Sivraj	Indoors	Restaraunt	Robot	Restaraunt Collborative Robot	Deliver food to customers in restaraunt	Algorithm	Robot path planning and obstacle avoidance algorithms
[34]	2019	Design of a Low-Cost Indoor Navigation System for Food Delivery Robot Based on Multi-Sensor Information Fusion	Sun, YL; et. al.	Indoors	Restaraunt	Robot	Restaraunt Robot	Meal delivery robot	Algorithm	Multi-source information fusion Robot Positioning Algorithm
[35]	2010	A new Automated Food Delivery System using autonomous track guided centre-wheel drive robot	YongChai Tan; et. al.	Indoors	Restaraunt	Robot	Restaraunt Robot	Automated Food Delivery to Customers	Design	System Design
[36]	2015	Towards finger gaming humanoid robot: mechanism and perception development	Lin, CY; et. al.	Indoors	Restaraunt	Robot	Restaraunt Robot for Entertainment	Play games with restaraunt customers	Prototype	Finger Game Playing Humanoid Robot
[37]	2018	Research on Moving Trajectory of Dining Robot Based on Matlab Simulation Technology	Kong, DP; et. al.	Indoors	Restaraunt	Robot	Takeaway Service Robot	Service Takeaway orders	Simulation	Robot Trajectory Simulation
[38]	2018	A technology acceptance model for the perception of restaurant service robots for trust, interactivity, and output quality	Lee, WH; et. al.	Indoors	Restaraunt	Robot	Restaraunt Robots	Robot use in order taking and serving	Survey	Survey of Restaraunt Manager’s Acceptance of Robot Use
[39]	2019	State of the art - Automated micro-vehicles for urban logistics	Baum, L; et. al.	Outdoors	Last Mile Delivery	AMV	Automated Micro Vehicles	Last mile delivery of supplies to homes	Review	Review Article
[40]	2017	Defending Against Intrusion of Malicious UAVs with Networked UAV Defense Swarms	M. R. Brust; et. al.	Outdoors	Last Mile Delivery	Drone	Networked Drone Swarms	Defend against malicious drone attacks	Algorithm	UAV defense system Algorithm
[41]	2017	Automated aerial suspended cargo delivery through reinforcement learning	Faust, A; et. al.	Outdoors	Last Mile Delivery	Drone	Cargo Delivery Drone	Deliver cargo such as food, medical supplies etc.	Algorithm	Trajectory Planning Algorithm
[42]	2019	Recording Provenance of Food Delivery Using IoT, Semantics and Business Blockchain Networks	M. Markovic; et. al.	Outdoors	Last Mile Delivery	IoT	IoT System	Monitoring food deliveries, safety of perishables in food supply chain	Design	Information System Architecture

A tabulation of articles on the dimensions of technology, and, the nature of artefact contributed by the study, is presented in Table 2. This analysis reveals that a majority of the studies are on motion planning of mobile robots. Few studies also explore design, simulation and cloud based software system design.

A tabulation of articles on the dimensions of technology, and, application context, is presented in Table 3. This analysis reveals that a majority of the studies are focused on indoor robots. Within indoor robot studies, six are in the restaurant industry, three in hospitals, and, one in a home setting. Of the four studies that are in an outdoor setting, two explore motion planning for robots and drones, one discusses a cloud based platform and one is a review of the use of automated-micro-vehicles in food delivery.

In summary, we find that extant research is focused on the use of robots in serving food in indoor settings like restaurants, hospitals and homes. In a singular study, Baum *et al.* [39] provide a comprehensive review of the use of Automated Micro Vehicles (AMVs), in last mile delivery of food and other supplies. While the technical feasibility of several of these automated vehicles has been established, there remain multiple challenges in them becoming operational and effective immediately.

Firstly, there are concerns around legislative and regulatory hurdles as well as community acceptance. For instance, San Francisco has banned all food delivery rovers that use the side walk [43]. Secondly, since the rovers use side-walks, they have to operate at low speeds which limits their ability to serve a large population in a reasonable amount of time. Thirdly, the rovers have small payload capacity which limits their ability to serve multiple customers in a single run. Fourthly, as, a United States Department of Agriculture (USDA) map of access to grocery stores reveals, a significant part of the US population does not live close to a grocery store (Appendix A). With a population so widely spread out, food delivery robots do not seem well suited for the purposes of distributing food at community scale. Finally, in the context of a pandemic, rovers that require interaction with customers, may themselves become a vector for contagion in a community. Because of these reasons, the use of delivery rovers has remained in experimental stages, and, limited to confined spaces like college campuses [44]. Thus, our literature review suggests that there is a lack of viable alternative models for delivering food and other essential supplies to all citizens in a safe, hygienic, and, affordable manner, during pandemics. To fill this need, we propose a ’Robotic Drive Through System’ (RDS) for distributing essentials in a fair and equitable manner. The system is designed to operate autonomously, without any human involvement, so as to prevent contagion. The order management system layer that oversees the intake and fulfillment of orders is designed to prevent hoarding, price-gouging and potential welfare fraud.

We start by presenting a conceptual model of the RDS in Section V. We next conduct a detailed analysis of the potential for automation in Section VI. Subsequently, in Section VII, we delineate the components of the proposed system, highlighting those within, and those out of scope, of the current study. In Section VIII, we lay out the system design process. A detailed design of the system components is presented in Section IX. These designs of the RDS components are used to perform a digital simulation, which is presented in Section X. In Section XI we layout the design of the cyber-physical layer.

Having designed and simulated the cyber-physical layers of the RDS system, we next present an overview of the information systems layer in Section XII. In here, we also present a detailed design of the end user app, and, of the order management system, which manages the intake and fulfillment of orders.

We next conduct a cycle time and throughput analysis of the complete system in Section XIII. In order to evaluate and validate sustained reliable operation of the system over long periods of time, a duty cycle analysis of the RDS robot was conducted in Section XIV. Finally, in Section XV, we present a comprehensive mapping of stakeholder requirements to the functional features of the system components. We note the limitations of the study in Section XVI and present our conclusions in Section XVII.

## V. Robotic Drive Through System

**FIGURE 7.**
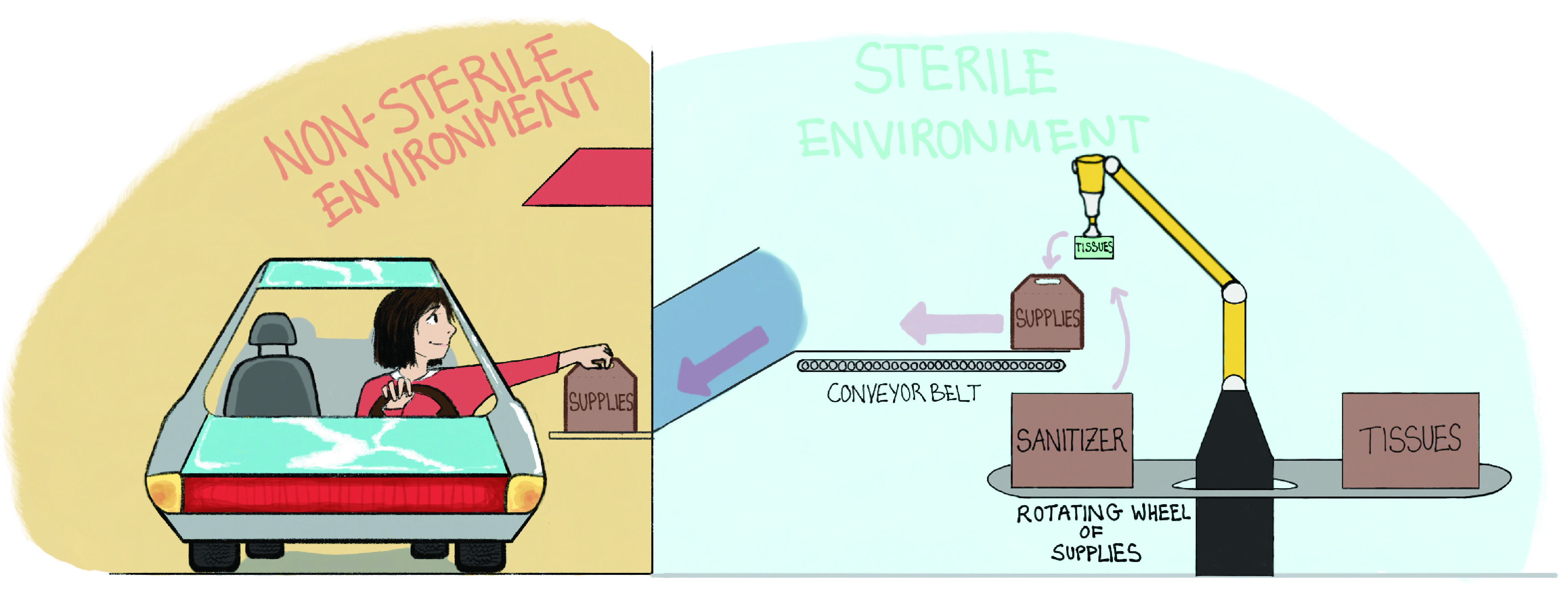
Artistic rendering of Robotic Drive Through System.

**FIGURE 8.**
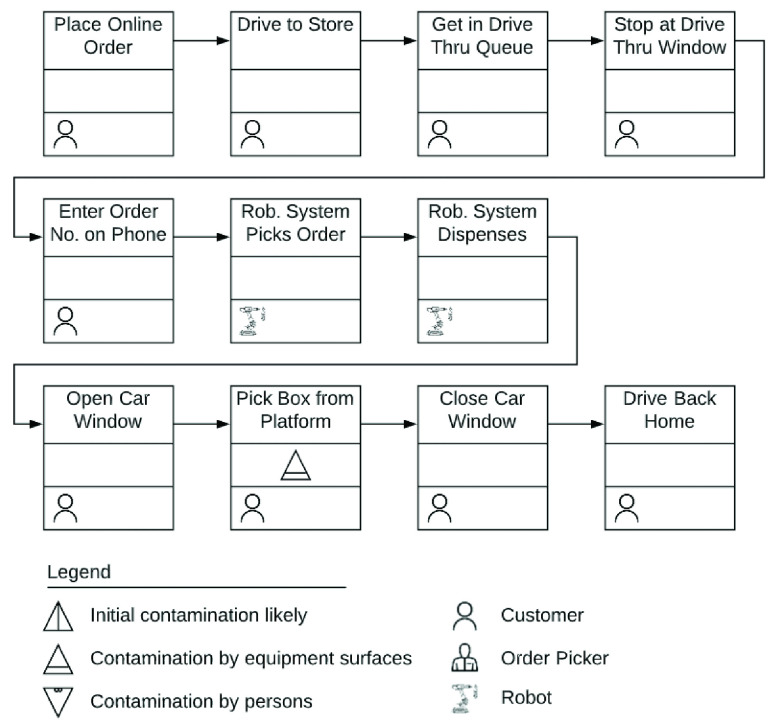
Process flow of drive-through grocery pick up.

Our proposed solution is a robotic cyber-physical system, which packs customer orders for food or essential supplies in a box, and dispenses it through a drive-through window. The customer takes her box from a pick-up platform and drives out of the counter area. The system is completely autonomous without any human in the loop. An artistic rendering is presented in Figure 7. A close look at the process flow diagram in Figure 8 clearly shows that the risk of contagion is nearly eliminated in the proposed system.

**FIGURE 9.**
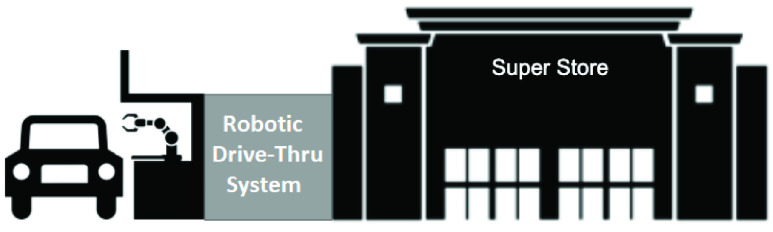
Drive through pick up from store.

**FIGURE 10.**
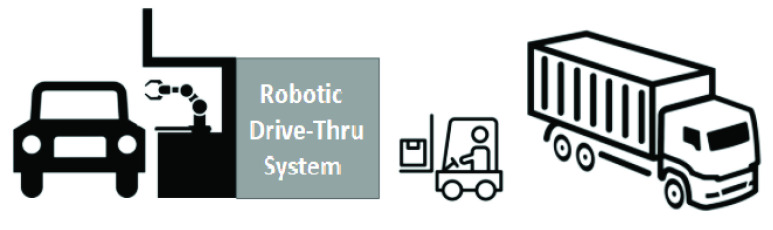
Drive through pick up from standalone distribution center.

The RDS has been designed to be modular and can be part of an existing operation such as a super-market, a convenience store or a food bank. It can also exist as a stand-alone mobile unit which can be deployed across multiple locations as needs change over time. An embodiment of the system when integrated with an existing store is presented in Figure 9. A standalone embodiment is presented in Figure 10. Having laid out the broad conceptual framework for the RDS, we evaluate the potential for automation, in Section VI.

## VI. Automation Evaluation

To evaluate the potential for automating RDS system operations, we start by specifying the sequence of steps to be executed. Because the RDS, in effect, replaces a grocery store order-picker, we consider the tasks completed by him in fulfilling an online order, as our starting point. The order fulfillment process starts with a customer order being assigned to an order-picker. The order-picker makes a tour of the store, collecting items that are on the list. If the order-picker does not find an item on the list, he may provide the customer an option to substitute it with another item. Finally, the order is bagged and delivered to the customer, either at home, or, on the curb.

While the tasks specified above are effortless for an able-bodied human, they might pose varying degrees of difficulty to a robot. For instance, mimicking the dexterity and versatility of a human hand in gripping and handling a wide range of objects, remains an unsolved challenge in robotics. Especially, food items which are soft, limp and of irregular shape, are not amenable to being gripped by the first generation of robotic hands which follow a ‘hard gripping’ approach. Another dimension on which robots are not as capable as humans is he ability to recognize items. However, as robots become increasingly interpretive technologies [46], they are begining to acquire almost human like abilities of cognition with sufficient training. On the other hand, robots may be better than humans on dimensions like repeatability, accuracy, and, sustained operation without errors.

Thus, it is not immediately clear which steps are viable for robotic automation. To evaluate each step individually, we consulted with industrial robotics automation experts in the areas of robotic grippers, machine vision, material handling and pick and place operations. These experts have between 10–30 years of experience designing, validating, implementing and supporting robotic automation solutions in the retail and food processing industries.

The panel of experts was presented with a description of the steps in order fulfillment. For each step, we considered how a human operator and a robot would execute it. In addition, a judgment was made whether the step could be automated within reasonable costs, while satisfying process constraints and stakeholder requirements. For each step, we also ascertained whether a robot or a human would be superior in executing it.

Results of this detailed analysis are summarized in Table 5. A careful look at this table reveals that most RDS steps are good candidates for robotic automation. Some of the tasks that seem difficult to automate, are, the removal of shrink wrap packaging from pallets, and, the opening of boxes. However, these tasks are upstream of order-assembly, and, remain out of scope of this study.

**TABLE 5:** Steps for Customer Order Fulfillment

No.	Operation	Step(s)	Req. #	Manual	Automated	Ease of Automation
1	Receive Goods	Receive pallets from delivery truck	1.1	Human Operator + Fork Lift	Autonomous Truck Unloading System	Difficult (Uncommon but Emerging application area)
		Store pallets in warehouse	1.2	Human Operator + Fork Lift	Autonomous Forklift System	Medium (Emerging application area)
2	Depalletize	Retrieve pallets from warehouse	2.1	Human Operator + Fork Lift	Autonomous Forklift System	Medium (Emerging application area)
		Unwrap plastic covers	2.2	Human uses sharp instrument to remove shrink wrap and other plastic covering on a pallet.	This is a non-trivial task for a robot*	Medium (Automated solutions exist)
		Transfer pallet to depalletizing work-cell	2.3	Human Operator + Fork Lift	Autonomous Forklift System	Medium (Emerging application area)
		Depalletize	2.4	Human Operator depalletizes pallet into individual boxes	Robotic Depalletizer	Easy (Mature Technology)
3	Make items Available for Picking	Open box	3.1	Human uses sharp instrument to open up box	This is a non-trivial task for a robot*	Medium (Automated solutions exist)
		Place item in store shelf or RDS tote	3.2	Human operator places items on store shelf	Robotic pick and place (Robot transfers items from box into RDS tote)	Easy (Mature Technology)
		Transfer and place tote in RDS work-cell	3.3	Not applicable	Tote is conveyed to and positioned inside work-cell using typical conveyor system or AGV	Easy (Mature Technology)
4	Pick Order	Identify items to be picked	4.1	Order-picker browses list of items in order	Robot receives order from order management system.	Easy
		Go to next item on list	4.2	Order-picker goes to store shelf with item	Tote with next item indexes in front of robot	Easy (Mature Technology)
		Find item	4.3	Order-picker finds item on store shelf	Robotic machine vision identifies within pile of items,an item with an easy to grab surface	Medium
		Pick item	4.4	Order-picker picks item	Robotic gripper picks item	Medium
		Place item	4.5	Order-picker places item in bag	Robot places item in order box	Easy (Mature Technology)
5	Pack Order	Close order box	5.1	Order-picker closes bag	Box sealer closes order box	Easy (Mature Technology)
6	Deliver Order	Deliver order box OR Dispense order box	6.1	Delivery operator delivers order	Dispensing mechanism drops order box on pick -up platform	Easy (Mature Technology)
		Customer picks order	6.2	Customer picks order at door step	Customer picks order box from drive through window	Not Applicable
			6.3	Not Applicable	Drive-through-pick-up platform self-disinfects	Easy

Another outcome of this detailed analysis was a natural coalescing of steps into logical groups, including, Receiving goods; Depalletizing; Making items available for picking; Picking an order; Packing an order; and, Delivering an order. We use this understanding to delineate the broad components that constitute the RDS. These components are discussed next, in section VII.

## VII. Components of Robotic Drive Through System

**FIGURE 11.**
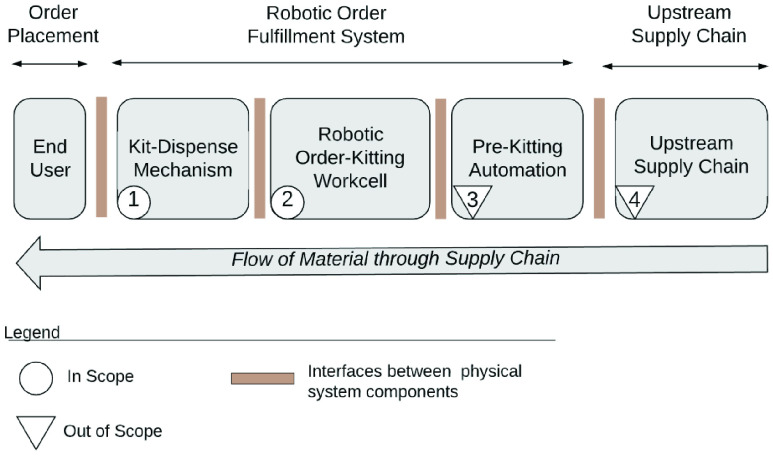
Components of robotic drive through system (RDS).

The RDS can be part of an existing operation (Ex: a super market or a food bank) (Figure 9), or a stand-alone operation (Figure 10). In either case, it is part of a larger system consisting of *upstream* and *downstream* sub-systems. A ’system of systems’ view is presented in Figure 11, in which, we also identify the sub-systems that are in and out of the scope of this study.

*Upstream* supply chain processes provision items for the RDS. While these processes (Component 4) are a critical enabler for the successful operation of the RDS, they are out of the scope of this study. *Downstream*, the orders may be delivered to customers through a variety of mechanisms such as autonomous delivery vehicles or robotic rovers. In the embodiment presented in this paper, downstream delivery is achieved by customers picking up their orders from a drive through window, as illustrated in Figure 7.

The RDS itself consists of two broad sub-systems: (a) order-placement, and, (b) order-fulfillment. In order-placement, a demand is placed on the RDS by the customer through an end-user app. A detailed design of this app is presented in Section XII. In order-fulfillment, the order is prepared by the RDS and dispensed to the customer. Order-fulfillment in turn consists of three distinct parts: (a) Pre-kitting operations, (b) Order-kitting, and, (c) Order-Dispensing.

The Pre-kitting operations (Component 3) bring the items from storage and make them available in the robotic work-cell. This includes steps such as receiving deliveries, storing and managing inventory, retrieving inventory from storage, de-palletizing, opening boxes, transferring items into appropriate containers, and, placing these containers inside the robotic work-cell. The exact equipment and mechanisms used in pre-kitting operations may vary across different configurations and deployments, and are out of the scope of this study.

In order-kitting (Component 2), a robot picks line items on a customer order and places them inside an order-box. The order-box is then sealed, labeled and conveyed for dispensing. In order-dispensing (Component 1), a transfer and dispense mechanism conveys the order-box out of a drive-through window and makes it available for customer pick-up. Both components 1 and 2 are within the scope of this study. Detailed designs are presented in Section IX. In Section VIII, we lay out the process followed to design the automation system.

## VIII. Robotic Automation Design Process

**FIGURE 12.**
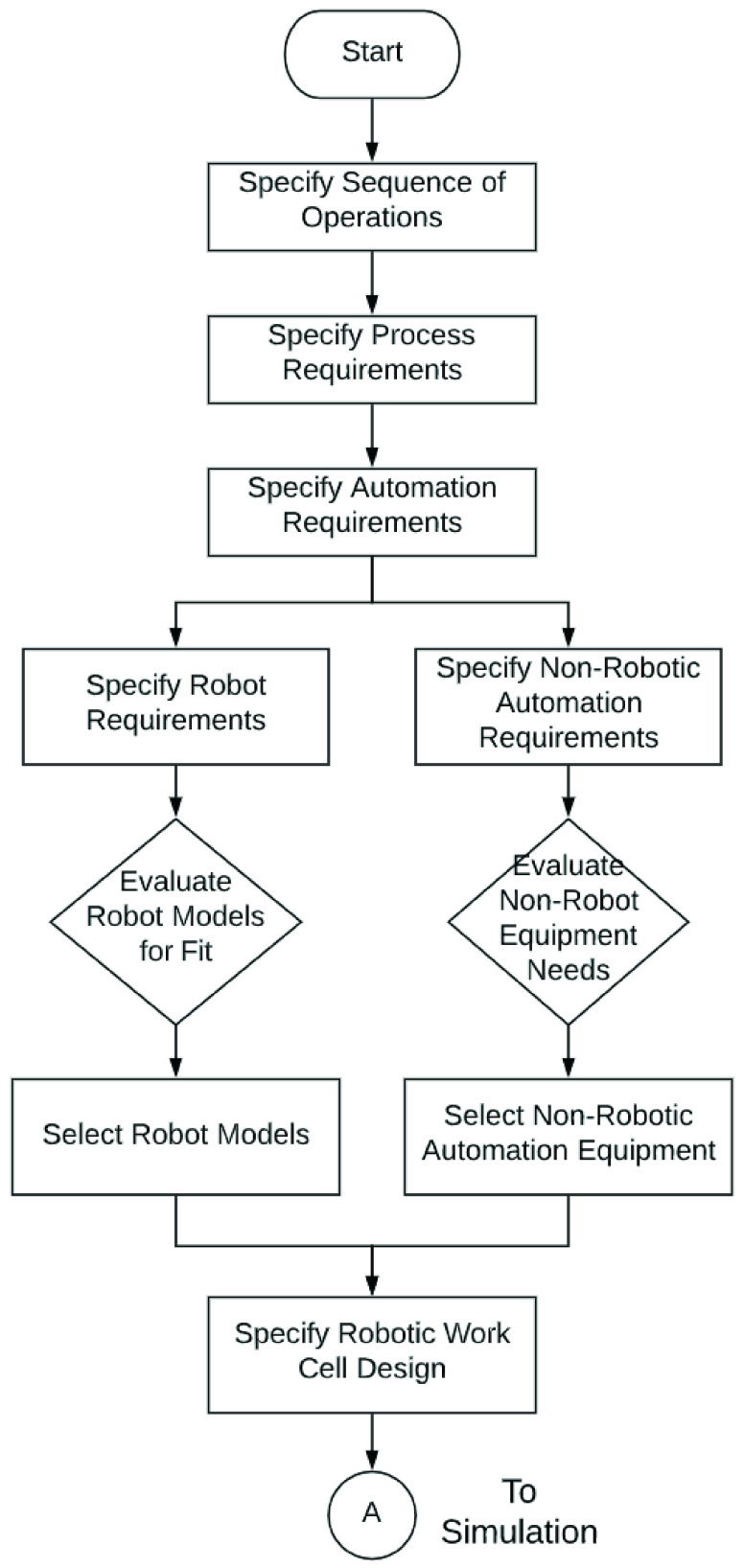
Automation design process.

Figure 12 lays out the steps followed in designing the RDS system. We use as our starting point, the order fulfillment steps laid out in Table 5. We start by specifying the detailed sequence of operations for each of the subsystems within the scope of this study (1 and 2 in Figure 11). Next, for each operation, we specify the process requirements under two categories - general and specific. Under general requirements we specify the standard requirements of reach, payload capacity, articulation, speed and precision. Under unique process requirements, we specify any requirements that are unique to a step. These process requirements are then translated into requirements for the robot and other automation equipment. These automation requirements provide the lens through which robot model and automation equipment options are evaluated and selected.

After selecting the robot, we evaluate and select the other non-robotic automation components of the work-cell, including the robotic end-of-arm gripper, conveyance mechanisms, order dispense mechanism and the order-box. Once the robot and automation equipment have been selected, we design and create digital twins of each of these components. We present the output of this design in Section IX.

## IX. Design of Robotic Drive Through System

We now present the design of the key components of the RDS. For each component, we present its requirements, the options evaluated, and, the design of the selected option.

### A. Robot Requirements and Selection

The central component of the RDS robotic work cell is the robot. The purpose of the robot is to pick the line items on a customer order and place them inside the customer order-box. The detailed sequence of operations for this pick and place operation is specified in Table 6. The general and specific process requirements are laid out in Tables 7 and 8 respectively. We presented these details to our panel of robotic experts who used them as a starting point to evaluate robot models for the RDS work-cell.

**TABLE 6:** Sequence of Operations for Order Kitting

No.	Operation Step
1	Individual S.K.U.s are placed inside standard totes in a pile.
2	Totes with S.K.U.s are placed on a circular Turntable.
2	Turntable indexes the totes so that they take turns to be the ’pick-tote’ which is nearest to the front of the robot.
2	Robot’s machine vision camera identifies a surface that can be easily grabbed.
2	Robot moves vacuum gripper to surface and activates suction.
2	Robot picks item and moves to top of order-box.
2	Robot places item in box, releases it and retracts arm.
2	The turntable indexes to tote with next item in customer’s order list.
5	Items are picked and placed in order-box till all items in order have been placed in box.
6	Once all items in order have been placed in order-box, it is conveyed to box sealer and the next empty order box indexes in.

**TABLE 7:** General Process Requirements for Order Kitting Operation

Parameter	Requirement
Reach	Robot Work Envelope Should allow for reaching into tote as well as placing into order-box.
Payload	Robot should have sufficient payload capacity tooling and expected range of product (all under 2 Kg)
Articulation	Minimum six degrees of freedom to reach items randomly oriented in tote (X, Y,Z,W,P and R).
Speed	Speed of robot is important to not keep customer waiting. Speed of work-cell is also dependent on turntable rotating and processing time of vision.
Precision	High precision is not a key requirement - +/−3mm can be tolerated.

**TABLE 8:** Unique Process Requirements for Order Assembly Operation

No.	Requirement
1	Robot should be able to pick items placed and oriented randomly in a pile.
1	Robot should be able to place item inside the order-box.
1	Robot should have sufficient speed to ensure order assembly of up to 7 items under 35 seconds.
1	Versatile end of arm tooling to handle a variety of item types.

A careful look at the process requirements reveals that for order assembly, a robot should have high speed, sufficient reach and payload capacity, high repeatability, and, moderate precision. High speed is needed to minimize or eliminate wait times for customers while the order is being filled. A sufficient work envelope is needed to allow the robot to pick items from all corners of the item container and also be able to conveniently place them inside the order-box. Finally, the robot must have the payload capacity to be able to pick the heaviest items. High precision is not a consideration because the gripping surfaces of the items are sufficiently large for moderate precision to suffice.

**FIGURE 13.**
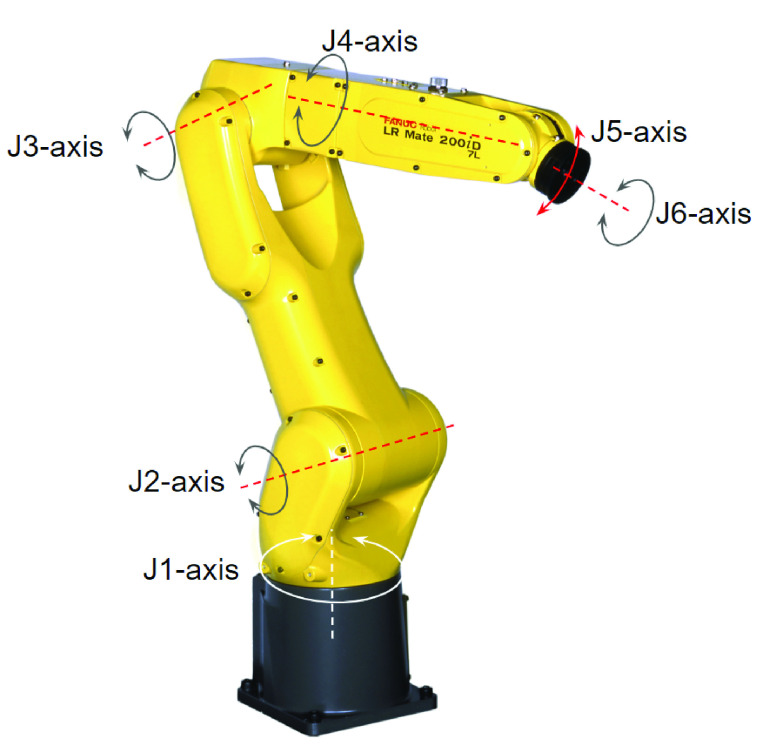
FANUC LR Mate 200iD/7L articulated robot.

**FIGURE 14.**
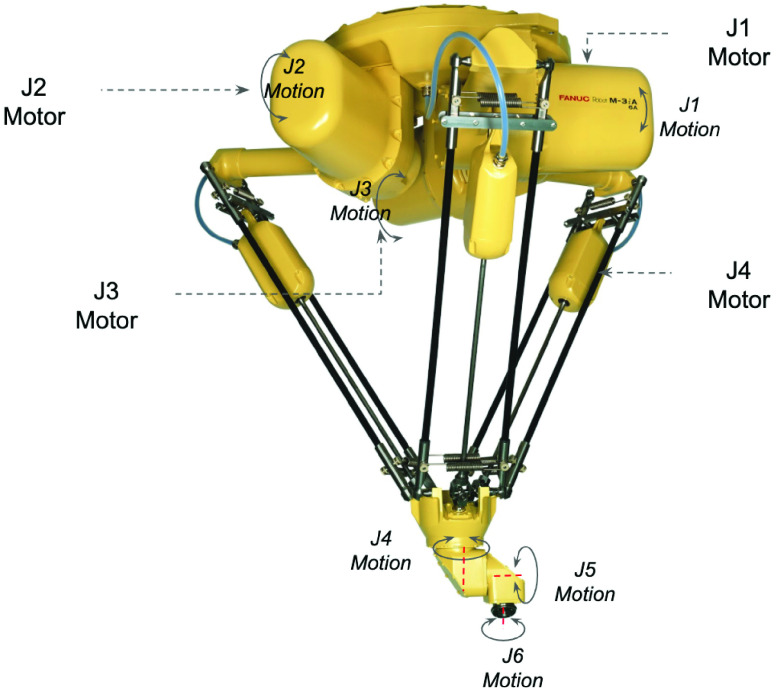
FANUC M-3iA/6A parallel linkage Delta robot.

Both, articulated robots (Figure 13), and, parallel linkage robots, also known as Delta robots (Figure 14), satisfy these requirements. Both of them have a payload capacity greater than 3 Kg which is well beyond the relatively light weight items that are in our assortment of products.

When considering speed, Delta robots are typically capable of achieving the highest speeds across all robot models. However, high speed and acceleration generate high inertia resulting in overshoot. Overshoot is the robot tool end point going beyond its desired destination point, especially at points where the direction of motion changes. For this reason, Delta robots may have to be operated at a speed below their theoretical maximum. While articulated robots have lower speed when compared to a Delta robot, they do have the advantages of a larger work envelope and more detailed articulation.

Another factor that is of interest is the duty cycle of the robot, which measures its ability to operate continuously at the specified speed and payload, without overheating of motors or premature mechanical failure of gears. The duty cycle of a robot is akin to a weightlifter lifting a weight, where only a certain amount of repetitions can be done before the weight lifter must rest to let his muscles recover. In a robot, if more heat is generated for a given motion than can be dissipated by the motor, the motor will eventually overheat and possibly fail. This can be managed by reducing the payload, slowing down the motion, or, adding rest periods to break down continuous motion.

Delta robots are especially suited for high-speed, high-duty cycle applications, because of their parallel linkages, which results in significantly less load on each motor. On the contrary, the mechanical linkages of an articulated robot are serial in nature, with each axes’ drive having to bear the load of all the subsequent axes’ drives. This in turn reduces the duty cycle of an articulated robot. An advantage of an articulated robot over a parallel linkage robot, is its compact design resulting in a smaller footprint. In addition, articulated robots are one of the lowest cost robot models.

A comparison of these two robot models on some key features is presented in Table 9. In summary, while Delta robots provide higher speeds, articulated robots have a lower cost and a smaller footprint. A comparison of the key features of articulated and Delta robots suggests that both models are suitable candidates for the robotic work-cell. However, as we take a closer look at some of the automation requirements, the Delta robot presents challenges that make it unsuitable for the RDS work-cell.

**TABLE 9:** Robot Requirements for RDS Work-Cell: A Comparison of LR-Mate and Delta Robots

Factor	6-axis articulated robot (FANUC LRMate200iD/7L)	Delta (FANUC M-3iA/6A Robot)
Reach	911 mm - sufficient reach for this operation.	1130 mm - insufficient vertical reach for this operation.
How many degrees of freedom	Six axes - sufficient for pick and place items	Six axes - sufficient for pick and place items
Payload	7 kg - sufficient payload capacity. Could not use 3 kg variant as it does not have sufficient reach.	6 Kg - sufficient payload capacity.
Repeatability/accuracy	±.01 mm - sufficient for this operation.	±.1 mm - sufficient for this operation.
Speed	[J1:370 °/sec, J2:310 °/sec, J3:410 °/sec, J4:550 °/sec, J5:545 °/sec, J6:1000 °/sec] For sustained operations, LR Mate is slower than Delta.	[J4:3500 °/sec] Delta is the fastest robot - Overshoot due to high speeds can imply that speeds lower than theoretical maximum are used.
Duty Cycle	For long term sustained operation, to maintain reliability, Motor Duty (overheat) and reducer life (Gear wear and tear) need to be managed. Robotic simulation software, such as ROBOGUIDE can be used to determine the robot speed and rest periods to maintain reliable performance over the life time of the robot	Delta robots are designed for continuous operations and therefore motor Duty and gear life is not a prominent consideration.
Cost	Moderately Low - One of the lowest cost robot model	High - Delta robots are significantly costlier than small 6-axis articulated robots like the LRMate200iD/7L. The structure holding the Delta robot can add upto 20% of the cost of the robot.
Footprint	Small	Large - Delta robots have an exclusive work space that cannot be shared
Non robotic system components	Mounting pedestals are relatively cheap	The overhead structure of a Delta robot can add upto 20% of robot cost.

First, because we plan to present items to the robot, in a pile inside a tote, the robot must be able to identify a surface which is properly exposed for easy gripping, from a pile of randomly oriented items. This can be achieved by a 3D vision sensor which needs to be mounted on, and, integrated with the robot. The overhead motor and linkage structure of a Delta robot, make it difficult or impossible to properly mount a 3D vision sensor. An articulated robot does not pose such challenges.

A second limitation of Delta robots is their inability to easily reach inside constrained spaces like totes and order boxes. The parallel linkage structure of Delta robots, increases the likelihood of running into the sides of the tote and the order-box. A slim armed articulated robot, on the other hand, is ideal for reaching into constrained spaces, without any interference. In addition, an articulated robot, has 6 axes of articulation which further enhances its ability to pick and place items in constrained spaces. Finally, an articulated robot allows for easy containment of robot tooling, wiring, and dress out equipment inside the internal structure of the robot. This helps minimize potential mechanical failure points in the work-cell, improving ongoing operational reliability.

All these factors make the articulated robot a lower cost, more compact, simpler, a more articulate, and, a more reliable option. This supports our objective of widespread deployment, and hence we selected an LR- Mate200iD/7L robot (Figure 13) for our work-cell. This robot model has just enough reach to pick randomly oriented items out of the tote and place them inside the customer order-box. A larger articulated robot model would perhaps allow more flexibility in the work-cell layout but come at a significantly higher cost.

### B. Gripper Requirements and Selection

The purpose of the end-of-arm gripper is to reliably pick items from a randomized pile and place them in the order-box. Hence, the gripper must be designed with two main factors in mind 1)the characteristics of items that are to be picked, and, 2) the manner in which those items will be presented to the robot.

All items in our assortment (hand-sanitizer bottle, hand-soap bottle, hand-gloves box, face-mask box, toilet paper roll) have non-porous surfaces which lend themselves to being picked by suction with a vacuum cup. Some of the surfaces, are somewhat curved, but the natural compliance of a soft cup will allow the suction cup to contour to the contact surface, allowing for robust gripping. Each of the items in our assortment was judged to have a flat enough, non-permeable surface, for a vacuum cup to generate sufficient suction to securely grip it.

Presenting the items in a pile, rather than in a neat stream of singulated items, raises the challenge of picking from a pile of randomly oriented items. This challenge is solved by the use of machine vision algorithms that are able to identify a surface that can be grabbed easily. The machine vision system will inherently guarantee that any product that will be picked is sufficiently near the top of the pile, as a large fraction of the product needs to be visible, to be found by the camera. This will ensure that the item that is attempted to be picked by the robot is not stuck under a pile of other items.

A typical two-finger mechanical gripper is also worth considering. Finger-based grippers need to have clearance on the sides of the item to grasp it. Due to the random, piled-up nature of part presentation, this condition may not always be satisfied. Additionally, a two finger gripper may not be effective in gripping a wide range of items with varying sizes and shapes. For instance, it might be challenging to grip cylindrical parts. Another challenge of finger grippers is the likelihood of them colliding with, and, damaging adjacent products in the tote. Because of these reasons, we do not consider mechanical finger grippers for our work-cell.

**FIGURE 15.**
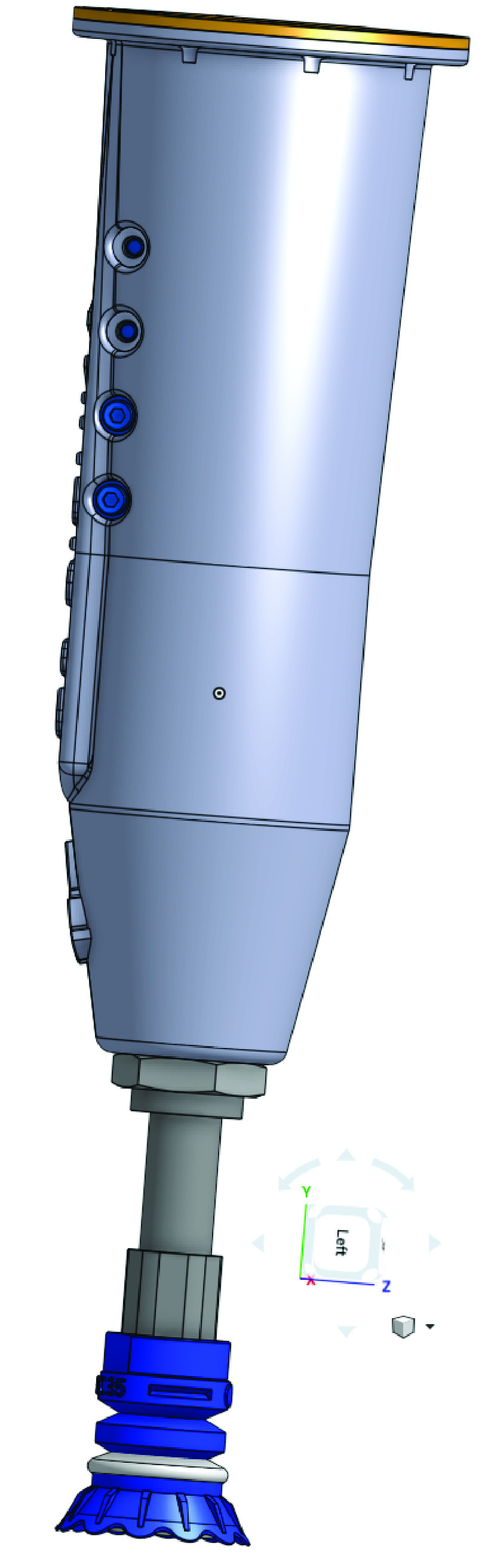
3D CAD of vacuum gripper: Side View.

**FIGURE 16.**
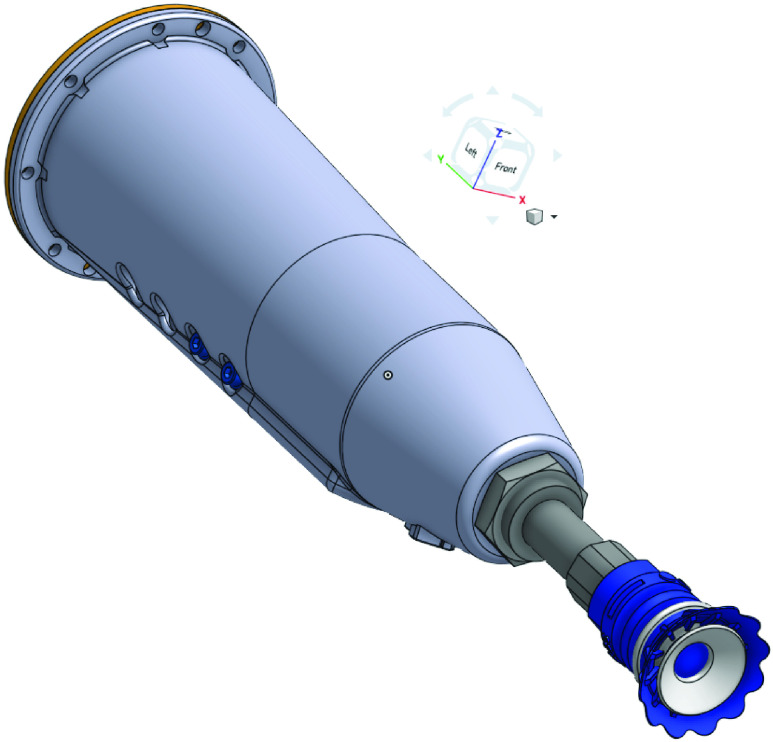
3D Cad of vacuum gripper: Front view.

Some of the other considerations for the end of arm tool, is to have a slim design to prevent interference with the sides of the tote or the order-box. Additionally, it is desirable to have a simple actuation mechanism to minimize the amount of dress-out equipment that might be needed on the end-effector. This minimization is useful in reducing the potential for mechanical failure. Because of all these factors, we selected a vacuum cup gripper, which is simple, versatile and integrates well with the rest of the RDS work-cell set up. Computer-Aided-Design (CAD) models of the gripper are presented in Figures 15 and 16.

### C. Item Indexing Mechanism Requirements and Selection

The item indexing mechanism is responsible for presenting the assortment of items to the robot for picking. This can be achieved through a wide variety of configurations. Two possible configurations are a linear conveyor or a circular turntable. The linear conveyor configuration requires more space because it needs one conveyor per SKU (stock keeping unit) item. As the assortment of items increases, the work-space needed may grow quickly and become untenable. In addition, a larger footprint, may pose difficulty in robot reach, necessitating the use of larger and costlier robots.

Because of these reasons, we selected a circular turn table configuration with a robot at the center doing the pick and place operation. The turntable allows for an efficient way to feed a repeatable set of items into the robot’s work area while allowing for a manageable footprint. It also allows for loading and unloading of totes, as the turntable indexes and rotates, without interrupting the normal operating cycle of the work-cell. The turntable configuration affords easy customization of customer orders as SKUs that are not part of an order can be easily skipped.

**FIGURE 17.**
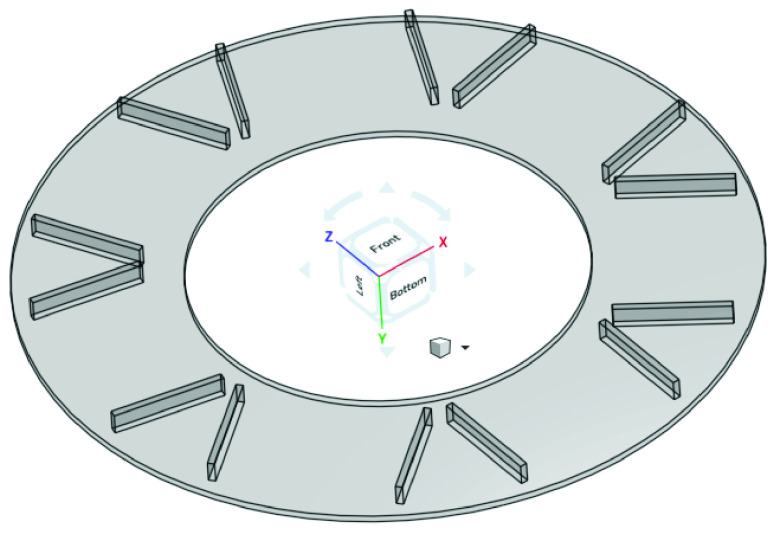
3D CAD of circular turntable for indexing and presenting SKUs.

A single turntable typically costs more than a single linear conveyor. However, the cost of multiple linear conveyors may add up to be higher. In addition, circular turntables are standard equipment that are easily available. Because of all these factors, we chose a circular turntable as our item indexing mechanism. A CAD model of the turntable is presented in Figure 17

### D. Tote Requirements and Selection

We have made a design choice to present the items in a pile to reduce space requirements, reduce system complexity, and, reduce the skill needed to operate the RDS. To keep the items in a pile, they need to be presented inside a container. We propose the use of a standard industrial grey tote. We chose this tote because it can hold a sufficient number of items, does not require lot of space, and allows for effective picking of items by the robot gripper. In addition, the use of the tote does not require high operator skills. To create the pile of items, the only step needed is to remove the items from their original packaging and place them inside the tote. Though this is likely to be a manual operation, depending on need, it may be possible to automate this step as well. Finally, using a industry standard tote offers the advantages of low cost, easy availability, operator familiarity and compatibility with other warehousing and material handling equipment.

### E. Order Box Requirements and Selection

The order-box is the container in which the customer receives her order. The robot needs to place customer order line items into this box. To afford easy access to the robot arm, the box should have as wide an opening as possible. Further, for reliable conveyance of the box to the drive through window, it should be rigid and uniform. In addition, the box should be tall enough to have sufficient space, and yet stable enough to not tip over. It should also be spill proof, and, low cost.

Two possible options are a box with lid cover, and, a box with flaps. A box with lid cover satisfies all the stated requirements, but requires additional custom automation to place the lid on the box. While a robot could place the lid, it may necessitate a larger, more expensive robot model to ensure reach. We therefore chose a box with flaps for our work-cell. A box with flaps does add the need for an additional box sealing machine to close it. However, box-sealers are readily available, off-the-shelf equipment. Hence we chose a box with flaps for delivering customer orders.

### F. Dispense Mechanism Requirements and Selection

The purpose of the dispense mechanism is to present the order-box to the customer, for pick up. It serves as the physical interface between the RDS system and the end customer, and hence is the only touch-point for possible germ transmission. For this reason, one of the key design requirements for the dispense mechanism is an ability to prevent the flow of germs. Another important requirement is easy physical integration with existing store operations, like a drive through window. It should also be made of components that are readily available, be low cost, and, be designed ergonomically for easy pick-up by customers.

Keeping all these factors in mind, we have proposed a simple slide that conveys the order-box to a pick-up counter outside the drive-through window. The counter has a revolving belt which turns after every order pick-up. The pick-up action can be accurately sensed using a weight sensor. During each revolution, the belt gets disinfected, minimizing the potential for transmission.

### G. Overall Robotic Drive Through System Design and Configuration

Till now we have listed out the requirements, and, designed the key components making up the RDS system. In addition to the requirements at the individual component level, there are requirements at the overall system level requirements. These are presented in Table 10. Some of the system level requirements, such as, low cost and small footprint, have already been satisfied as part of the design of individual components. Other requirements, like ease of integration with existing operations and IT systems, are at a larger system level, and not been discussed explicitly in this study. However, the design of components has taken into consideration requirements such as modulatiry and ease of integration.

**TABLE 10:** Requirements for Overal RDS System

No.	Requirement
1	Should be compact with a small footprint.
2	Should be low cost.
3	Should be easy to deploy.
4	Should be easy to operate and maintain.
5	Should be easy to integrate with upstream supply chain activities.
6	Should be easy to integrate with existing store IT systems.
7	Should be easy to integrate with drive through operations.
8	Should follow principles of reuse, recycle to the extent possible.
9	Should allow for reconfiguration to larger number of items, based on space availability.
10	Should be modular allowing for deployment as a stand alone unit, or integration with an existing operation such as a super market.
11	Should allow for deployment as a mobile unit which can be relocated to high priority areas.

**FIGURE 18.**
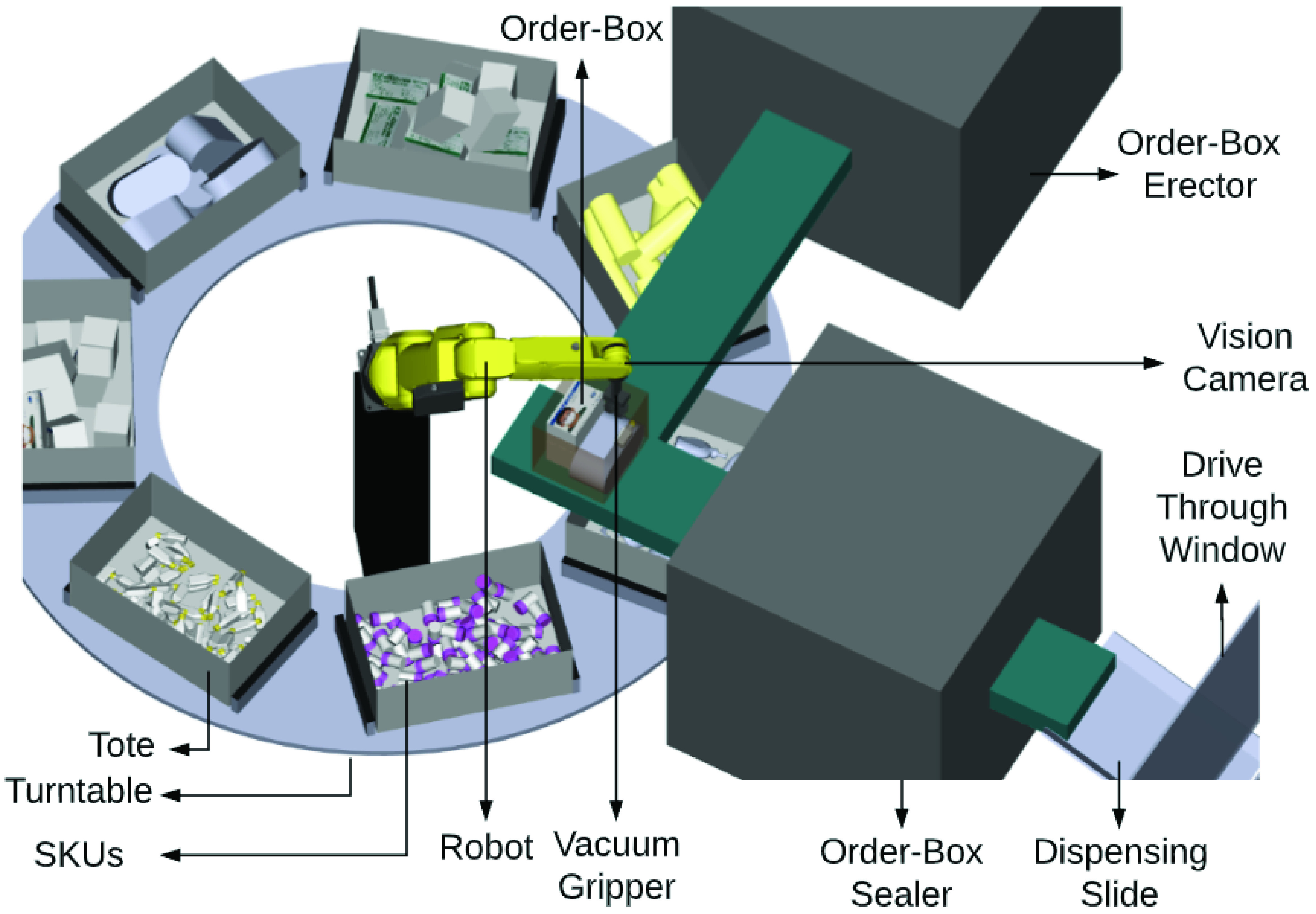
Digital twin of robotic work-cell in ROBOGUIDE.

**FIGURE 19.**
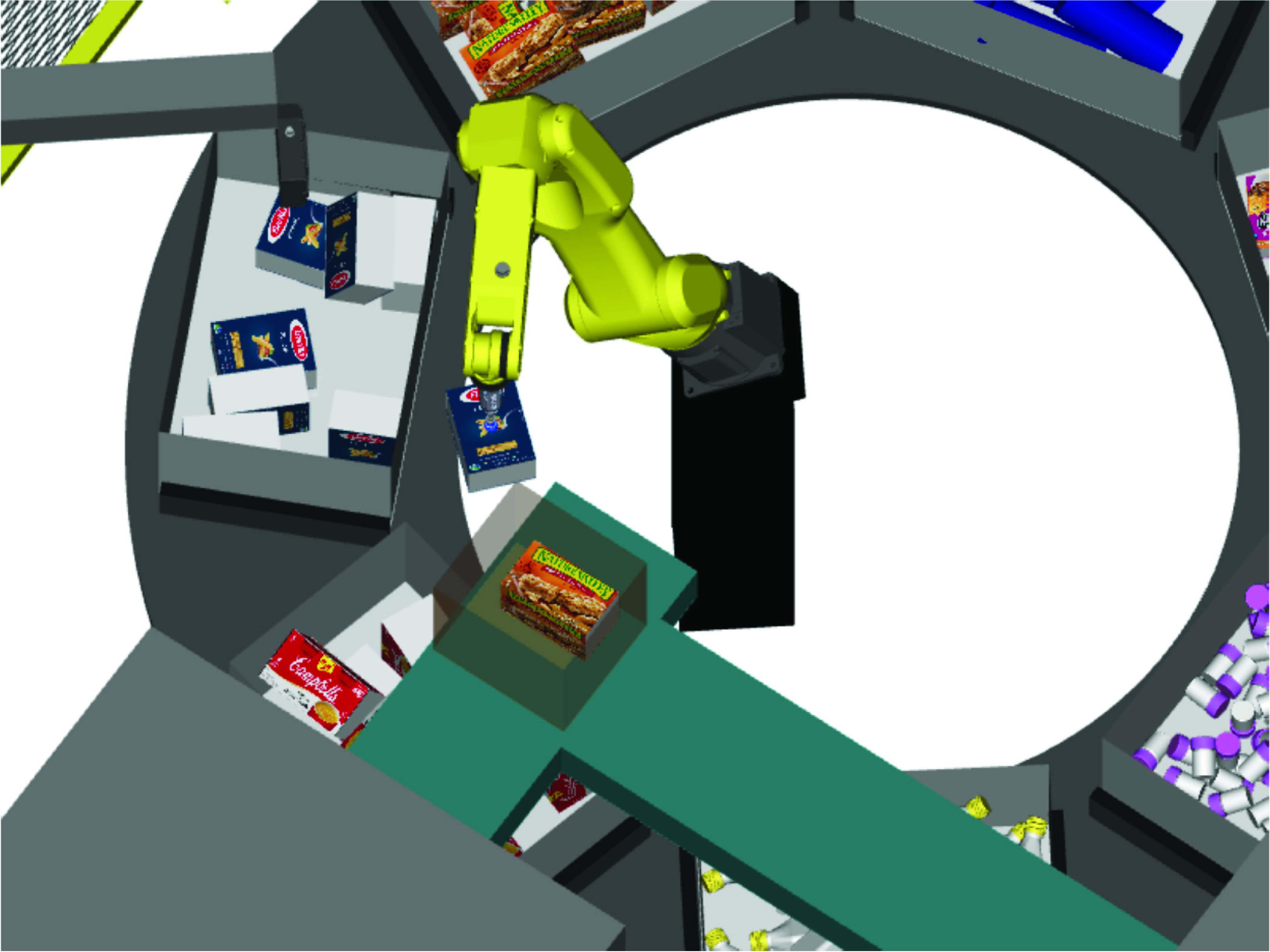
Digital twin of robotic work-cell for distributing food.

**FIGURE 20.**
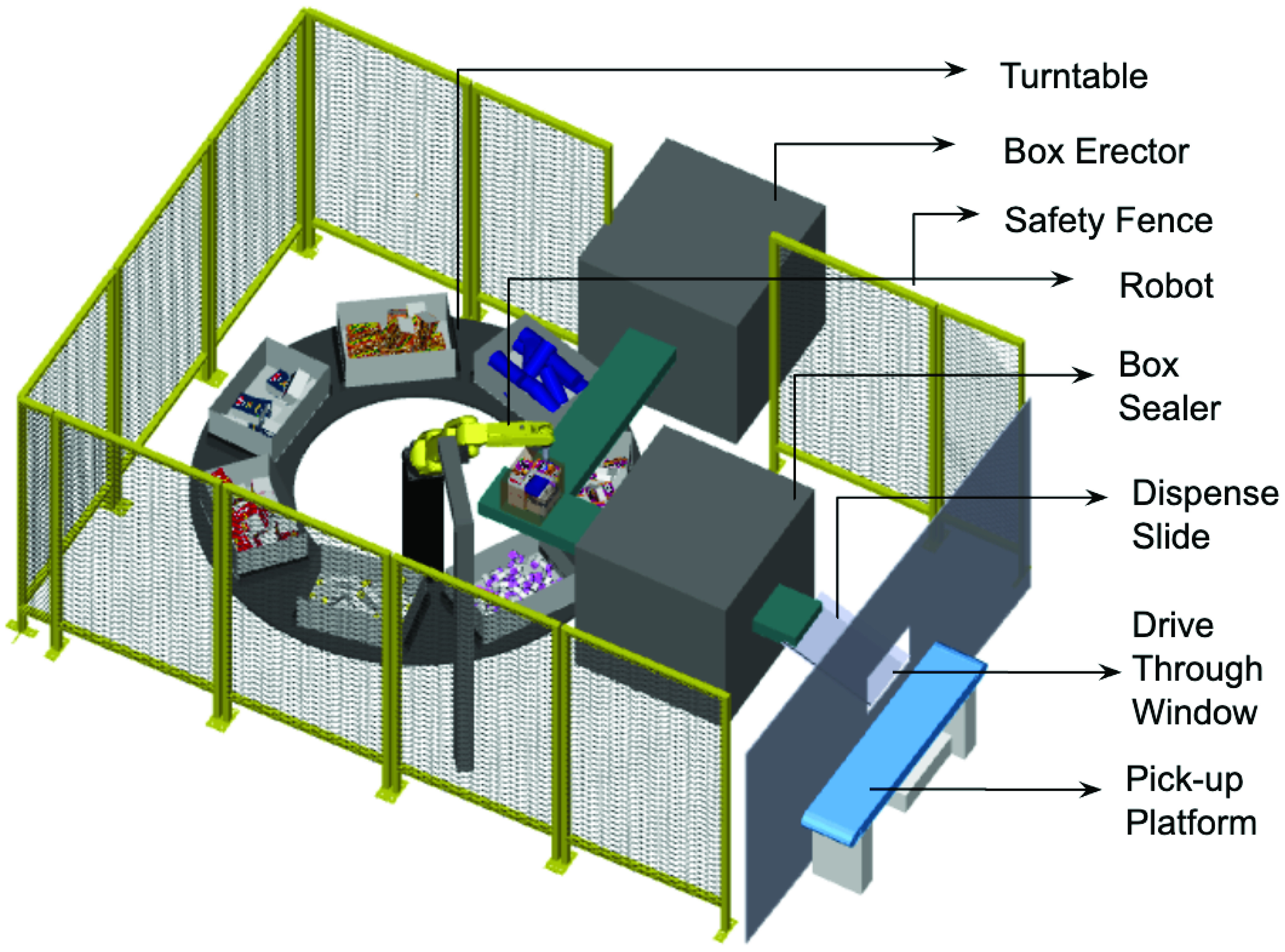
Layout of robotic work-cell for food distribution.

This completes our system design step. The individual component designs are now assembled together in a robotic simulation software, to create a digital twin model of the complete RDS system. This digital twin is presented in Figure 18, and serves as the input to robotic work-cell simulation which is discussed in Section X. The digital twin presented in Figure 18, is for picking essential supplies including hand sanitizer, tissue box, medicine bottle and hand soap. Another version of the work-cell was also simulated and validated for picking food items like granola bars, pasta box and canned soup, and its digital twin is presented in Figure 19. A zoomed out version of the work-cell is presented in Figure 20. As is clear from Figures 18, 19, and, 20, the system as designed is highly versatile and can be used for a wide variety of items. Making suitable changes to the end of arm tooling can further diversify the assortment of items that the sytem can handle.

## X. Simulation of Robotic Drive Through System

One of the objectives of this research project is to propose a solution that is *implementation ready* for deployment by organizations interested in distributing food and essentials to their communities. A critical consideration in automation solutions is sustained reliable operation over long periods of time. To ensure this, industrial automation solutions are subjected to ’end of life testing’ and ’accelerated life testing’. These tests require setting up physical prototypes and conducting dry run simulations to identify points of failure in the system. Long run testing is required because some failure points do not surface immediately. While it is possible to evaluate robotic automation solutions by setting up physical prototypes, it can be a time consuming and expensive process. In addition, each iteration of a physical system requires substantial time and can slow down the system development and validation process.

Because of these reasons, digital simulation is becoming the industry standard for validating robotic automation systems. Digital robotic simulation software tools allow us to create a digital-twin of the robotic work-cell, taking into consideration real-world constraints and process requirements, without the need and expense of setting up a physical prototype. This allows simulating the exact workings of a proposed automation solution, providing insights into its performance in a real-world deployment. Further, a digital simulation allows quick changes and fast iterations resulting in accelerated solution development and time-savings.

### A. ROBOGUIDE: Robotic Simulation Tool

A variety of robotic simulation tools are available. For example, RobotStudio from ABB, DELMIA from Dassault Systemes, Tecnomatix from Siemens and ROBOGUIDE from FANUC. Since we have used FANUC robots in the RDS system, we used FANUC’s proprietary simulation software, ROBOGUIDE. With virtual robots and work-cell models, as well as offline programming, ROBOGUIDE enables visualization of single and multi-robot work-cell layouts.

ROBOGUIDE allows the design and generation of three dimensional models of manufacturing work cells using included libraries with built-in models of all FANUC Robots, generic models of robot end-of-arm tooling (eg: vacumn gripper, mechanical gripper etc.), and, generic models of non-robotic components (e.g. conveyors, tables, platforms, fences). Components that are not available within ROBOGUIDE can be imported from external CAD environments. For instance, in our example work-cell setup in Figure 18, the turntable, tote, order-box and items have been designed in SOLIDWORKS and imported into ROBOGUIDE. This work-cell also uses in-built components such as robot controllers, IR vision cameras, conveyors and safety enclosures. A key component that is simulated in ROBOGUIDE is the robot controller. The robot controller is the computer that serves as the brain that controls the robot. It is important to note that when a robot controller is included in a ROBOGUIDE work cell, an exact replica of the controller software that controls an actual robot is loaded (Figure 22). The fact that the simulation software is run on an emulation of the real robot controller allows for near identical duplication of the motion performance between the virtual and the real robot. Further, several robots working in co-ordination, each under the control of a separate robot controller may be simulated within a work-cell. Close fidelity between the digital and real-world system helps to significantly reduce the risks for manufacturers before a physical installation.

**FIGURE 21.**
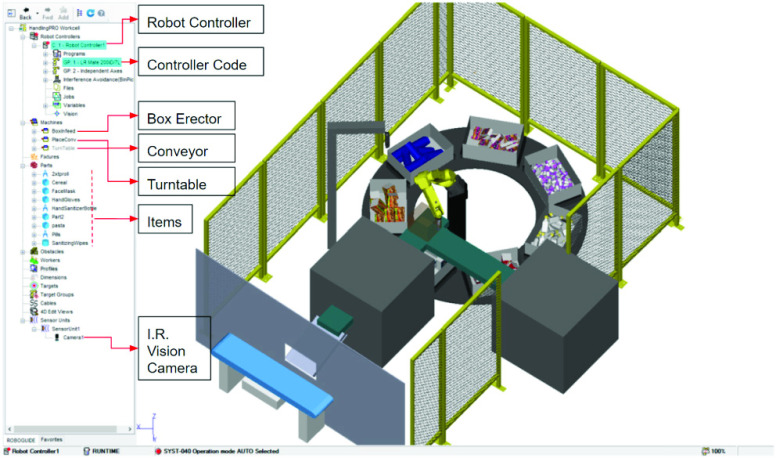
Work-cell setup structure in ROBOGUIDE simulation software.

**FIGURE 22.**
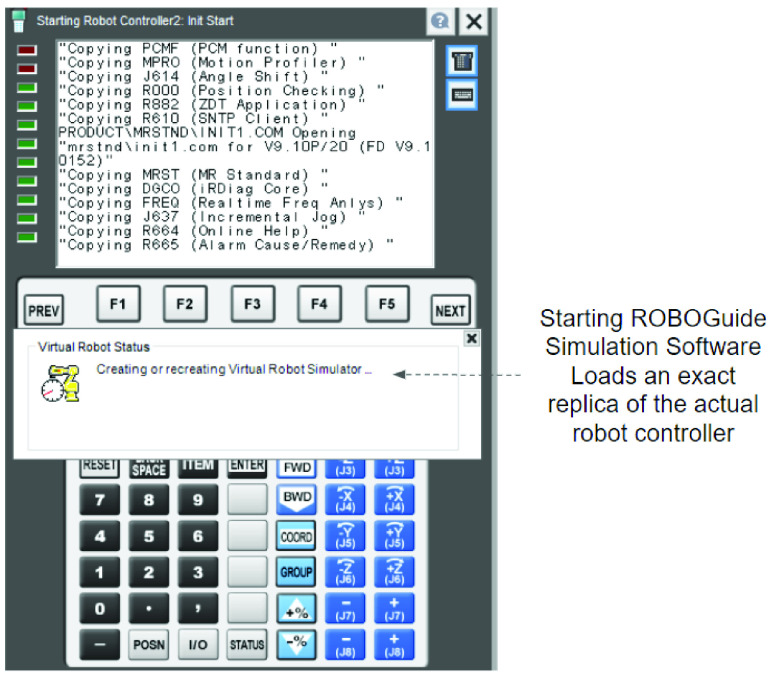
Virtual robot controller software.

**FIGURE 23.**
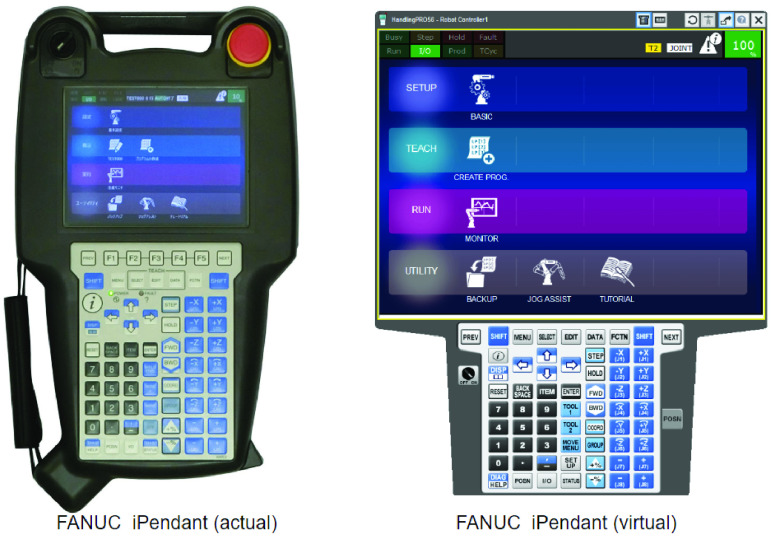
Physical teach pendant and virtual teach pendant.

**FIGURE 24.**
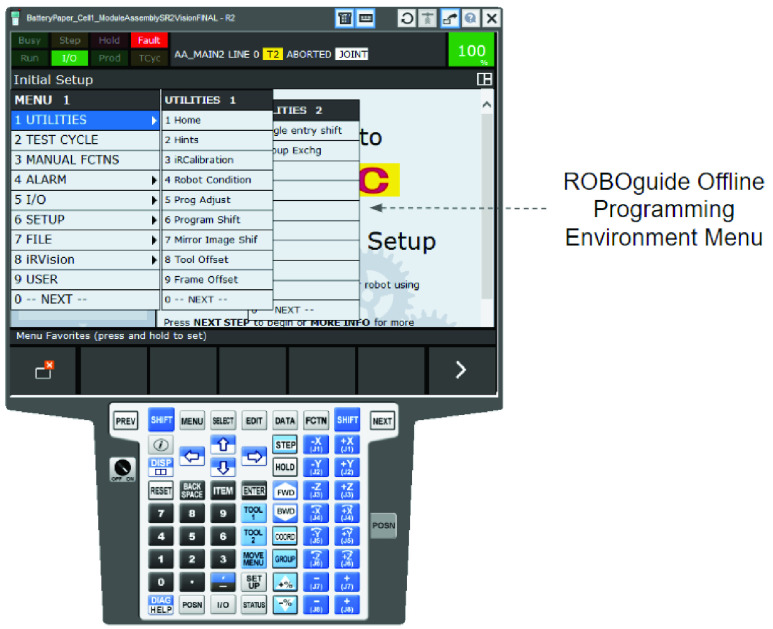
Off-Line robot programming environment menu.

**FIGURE 25.**
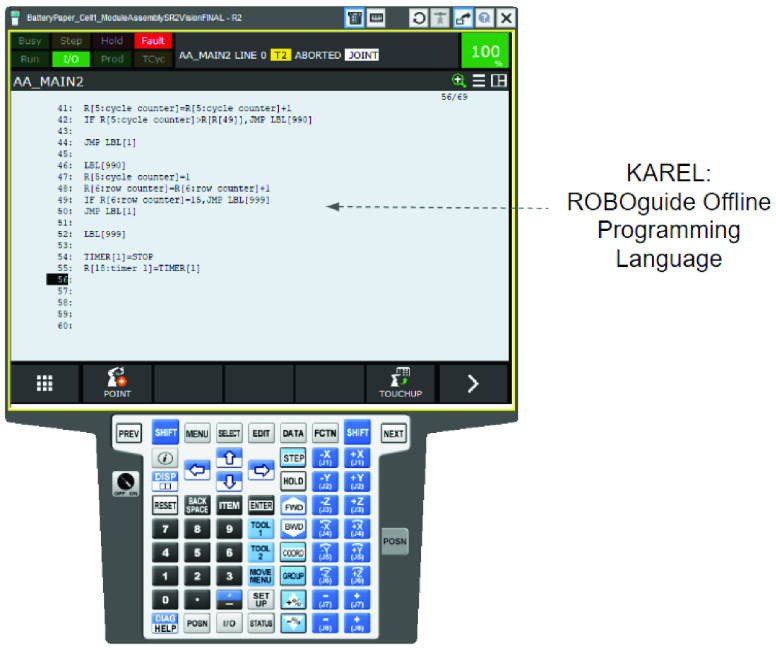
KAREL: ROBOGUIDE Off-line robot programming language.

Like most other robotic simulation software, ROBOGUIDE also provides an offline robot programming environment. The interface used to program the simulation is a virtual replica of the same teach pendant that would be used on an actual robot. An actual teach pendant, and, its virtual emulation in ROBOGUIDE, is illustrated in Fig. 23. Off-line programming can be done using a drop down menu environment which is an exact replica of the physical teach pendant (Fig. 24). In addition, for complex programming needs, a proprietary scripting language called Karel is also available (Fig. 25).

**FIGURE 26.**
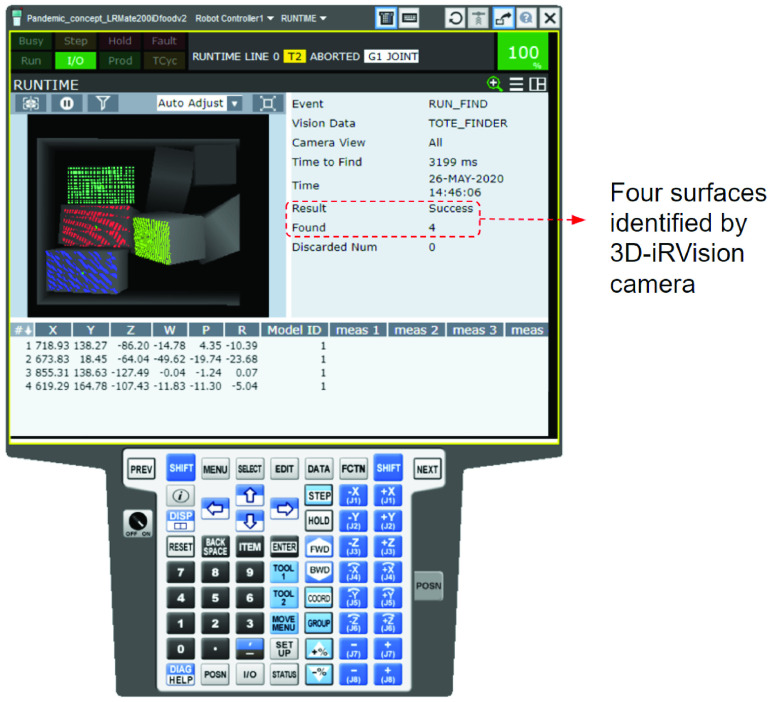
Off-Line simulation of ${i}$RVision camera functionality in ROBOGUIDE.

Apart from the robot controller, other components also replicate their real life properties. For instance, the motion of conveyors and the functioning of IR vision cameras replicates how they would operate in the real world. Fig. 26 shows the digital simulation of the IR vision camera finding an easy to grab surface within a pile of randomly oriented objects. In the illustrated simulation, the camera finds a surface to grab. Under the exception of it not finding a surface, the camera would flash a failure and send a signal to the tote to vibrate in order to shake the pile and reorient the items.

**FIGURE 27.**
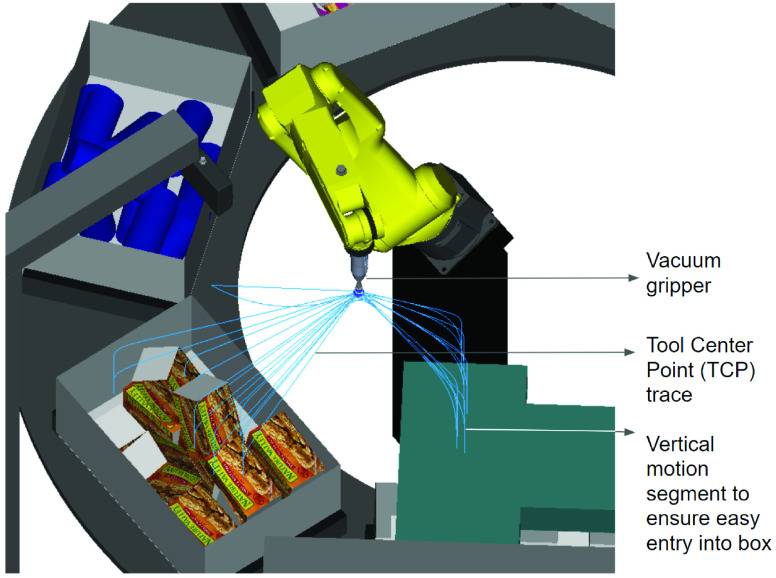
Tool path trace (TCP) of LRMate articulated robot during item pick and place in RDS work-cell.

In addition to simulating the behavior of the robot controller, ROBOGUIDE also simulates the mechanics and dynamics of the robot. The payload capacity of robots is respected and if exceeded, an error code is generated. The virtual programming environment also lets the user perform advanced analysis on the motion path via the ’Tool Center Point’ (TCP) trace, as shown in Figure 27. The TCP trace clearly illustrates how the motion path of the robot has been planned such that the robot enters the tote and the order-box in a vertical motion, so as to avoid any collisions. TCP trace can be used to verify clearance between robots and fixed components as well as show speed and acceleration of the robot’s tool center point. During the simulation of the robot program any collision that occurs between objects in the work-cell may be automatically reported. Cycle times can be calculated for the overall sequence of movements. In addition, the virtual environment provides the ability to perform duty cycle analysis as well as gear life analysis. This helps indicate the real-life effects of the virtual programmed path on the robot motors and gears.

### B. Simulation Method

**FIGURE 28.**
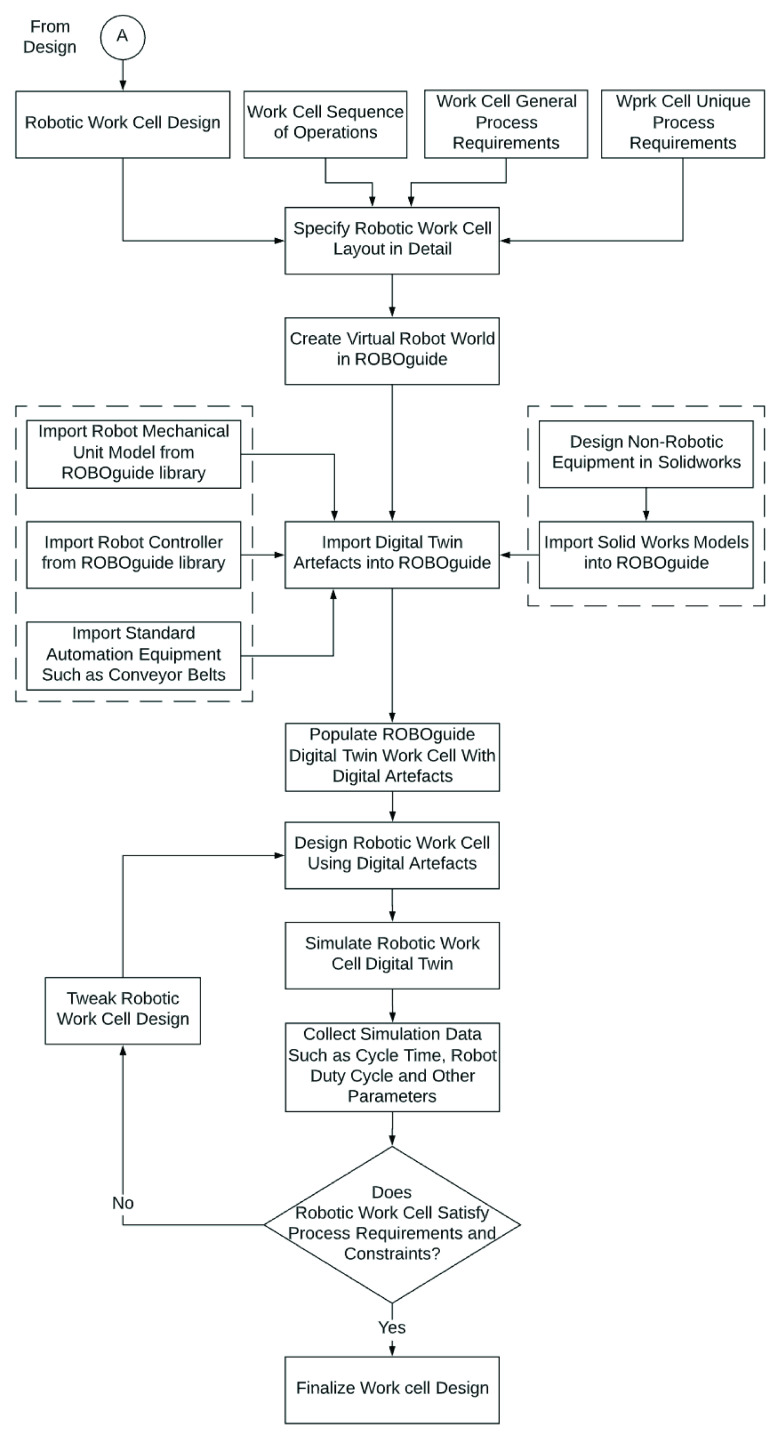
Robotic work-cell simulation process.

For the simulation phase, we used the work-cell design developed during the design phase in section IX as our starting point. In addition, we also reference the sequence of operations and the process requirements for each operation in setting up the simulation in ROBOGUIDE. A flowchart specifying the simulation process is presented in Fig. 28.

The first step was to create a virtual robot world in the ROBOGUIDE simulation software. We then imported in-built models of the FANUC robots from the ROBOGUIDE libraries. The models of the robot mechanical units emulate the exact dynamics of a robot system which ensures that all movements are feasible and realistic. From the ROBOGUIDE libraries, we imported the 3D CAD model of the vacuum gripper. We also imported standard components such as vision cameras, conveyors, stands, robot overhead structures, safety enclosures, and other components as needed in the work-cell. To develop 3D-CAD models of non-standard components such as work pieces, end-of-arm tooling, and other components required in building the work-cell, we used SOLIDWORKS CAD software. These 3D models were then imported from SOLIDWORKS into ROBOGUIDE.

The Robot model and 3D models of components were then used to construct layouts for the RDS work-cell in ROBOGUIDE. The robot was then taught positions and motion paths. Finally, the robot were programmed using the robot controller emulator contained within ROBOGUIDE. The robot virtual controller was programmed to meet the needs of the RDS work-cell as discussed in previous sections. Non-robotic automation aspects of the work-cell, such as items attaching to end-of-arm-tool when picked, and, detaching when released, were programmed and animated to interact with the robot to fully convey the operation of the cell. When necessary, motion of the robot was tuned to avoid interference with other work pieces, fixtures and components in the work-cell. Furthermore, robot location within the work-cell, as well as robot motion speeds, were tuned to depict a realistic cycle that takes into consideration sustained motor performance and longevity of the robot’s gears over long term operation. Gripper actuation times, were estimated based on consultations with application-specific experts who have on average two to three decades of experience designing robotic work-cells.

After the work-cell layout has been detailed out in ROBOGUIDE, we run the simulation. ROBOGUIDE emulates the mechanical behavior of the robot and components. In addition, it also emulates the behavior of the robot controller and program as it will play out in the real world. Robotic simulation software have now advanced to even emulate the movement of flexible components like cables and wires, which was not possible earlier. This level of emulation of the real-world results in reproduction of actual robot kinematics and motion performance, including, motion speed, motor duty, and gear life estimation of the robot at specified payloads. Additionally, because the simulation is a digital twin, it limits the robot to its realistic work envelope. This is critical as most robots have dead zones which they cannot reach.

Running the simulation allows us to detect collisions between robots and other objects, spot areas that are beyond the reach of the robot, simulate the payload capacity of the robot, simulate the execution of the software within the robot, simulate the dynamics of the robot, simulate the coordination of the movement of the robot with the rest of the non-robotic automation equipment within the work-cell, and evaluate and optimize time taken for the sequence of movements. We continue to iterate and refine the work-cell layout and robot program to optimize the work-cell for cycle time, robot duty cycle, robot gear life and power consumption. Once we are satisfied with the robotic work-cell operation, we finalize the work-cell design. This completes our simulation of the RDS.

## XI. The Cyber-Physical Layer

Industrial automation systems are cyber-physical in nature at progressively higher levels starting from a robot, to a robotic work-cell, to an assembly line, and, to the plant level. At each level, they are a composite of cyber, physical, and cyber-physical components. The physical layer consists of the mechanical elements such as robots and conveyors. The cyber layer consists of the software that orchestrates the automated operation of the physical layer. The cyber-physical layer consists of the process controllers, Programmable Logic Controllers (PLCs) and industrial computers on which the automation software resides and runs.

Considering the robot as a cyber-physical system, the robot mechanical unit makes up the physical layer, the robot program makes up the cyber layer and the robot controller constitutes the cyber-physical layer. The robot mechanical unit is controlled by the robot controller which is an industrial computer made of high-performance hardware and the latest advances in network communications, integrated ${i}$RVision, and motion control functions. In effect, the robot controller is the brain that controls the motion of the robot. The software that runs on the robot controller is typically written in a proprietary programming language. For instance, Karel is the programming language for Fanuc controllers. Usually, these programming languages also come with a range of libraries and functions that can be used readily for often-used standard functionalities. For instance, Fanuc’s programming environment contains over 250 different software functions for enhanced intelligence, motion and safety. With the increasing sophistication of these algorithms that are becoming available in robots, they are becoming increasingly interpretive and intelligent.

At the work-cell level, the physical layer consists of all the equipment making up the work-cell, including the robot. The cyber-physical layer consists of the robot controller and the process controllers for other automation equipment, including conveyors, AGVs, and, tool changers. Figure 30 illustrates the various controllers in the cyber-physical layer of our proposed RDS system. It consists of a master controller that controls the overall system (Component 5), the robot controller (Component 7), controller for the pre-kitting automation (Component 8), and the controller for the dispense mechanism (Component 6).

**FIGURE 29.**
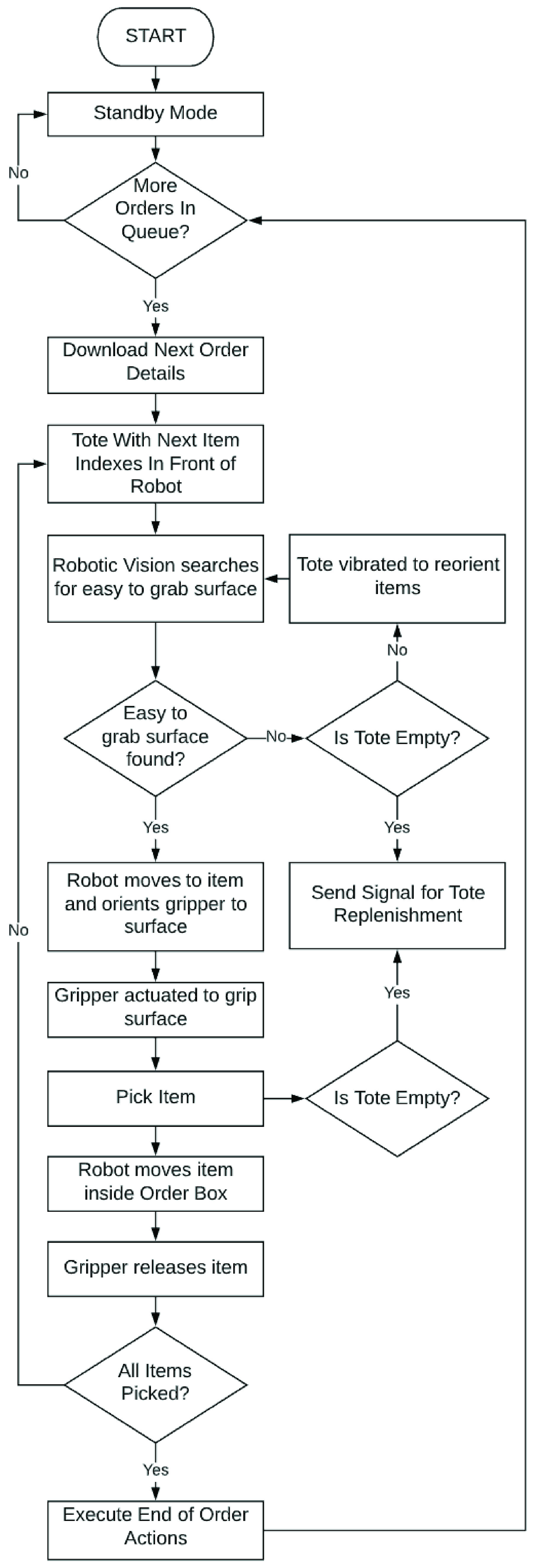
RDS control logic.

**FIGURE 30.**
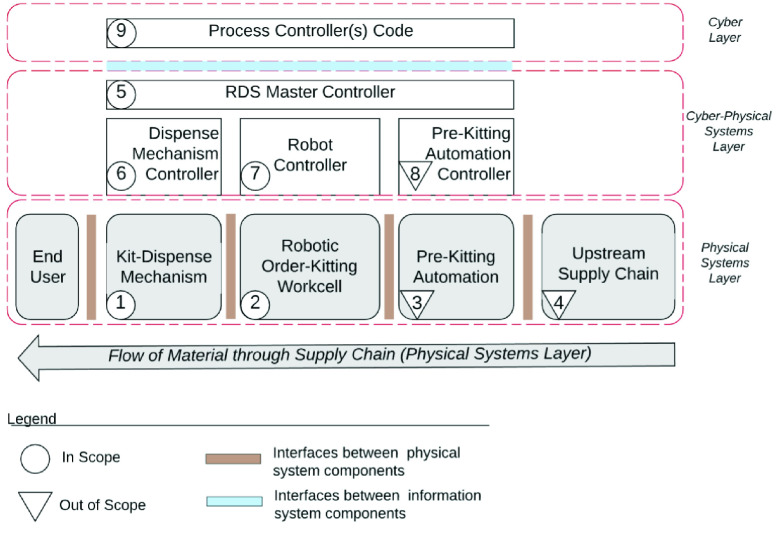
Cyber-physical layers of the robotic drive through system (RDS).

Finally, the cyber layer of the RDS, consists of the software that is loaded on the various controllers making up the cyber-physical layer of the RDS system. This software orchestrates the automated operation of the robot and other equipment within the RDS system. A flowchart detailing the RDS control logic is presented in Figure 29. This control logic was written using the Karel programming language in ROBOGUIDE’s offline programming environment. As pointed out in the description of the simulation process, this control logic is run on virtual emulators of the process controllers within the RDS system. This ensures that the final software thus developed, is ready for use in an actual system. It can be directly downloaded and used on the process controllers of an RDS system deployed in the field.

## XII. The Information Systems Layer

**FIGURE 31.**
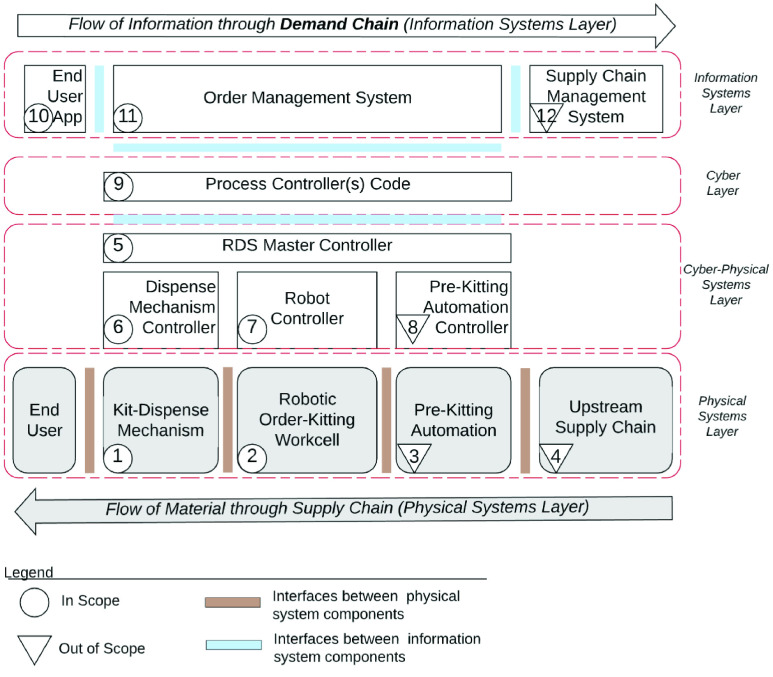
Cyber-physical and Information Layers of the Robotic Drive Through System (RDS).

The Cyber-layer of the RDS controls the automated operation of the physical layer consisting of the robot and other automation equipment. However, a customer does not interact directly with the cyber-layer of the RDS system, when placing orders. Customers place their demand through an information systems layer that sits on top of, and, communicates with the cyber-physical layer. This is illustrated in Figure 31.

The information system layer manages the flow of demand information from the customer to the RDS. It consists of an end user app, an order management system and a supply chain management system. The supply chain management system (component 12) is responsible for managing the upstream supply chain processes and is out of scope of this study. The end user app (component 10), and, the order management system (component 11) are within the scope of this study. In what follows, we present detailed designs of these components.

### A. End User App Design

**FIGURE 32.**
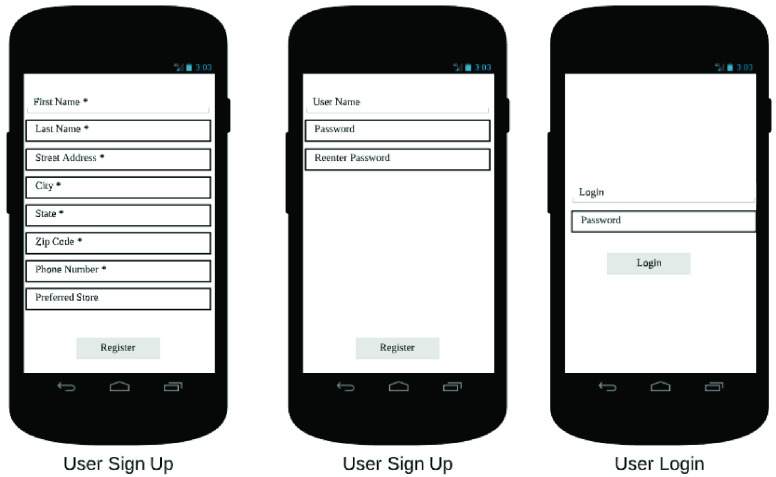
User sign-up.

**FIGURE 33.**
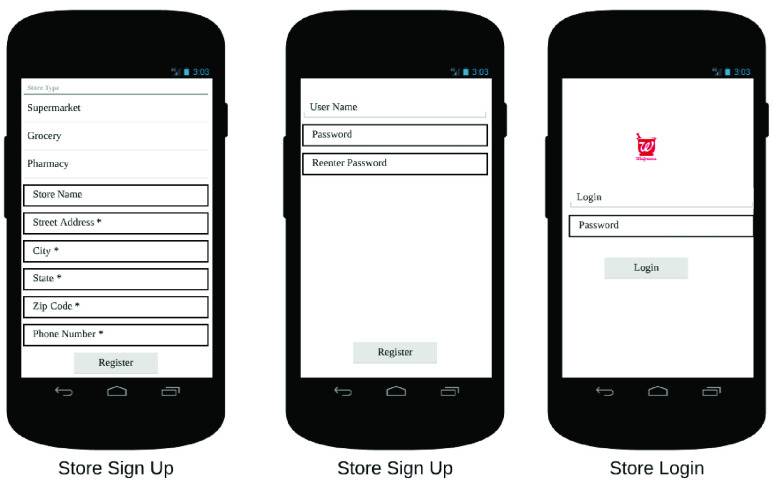
Store sign-up.

**FIGURE 34.**
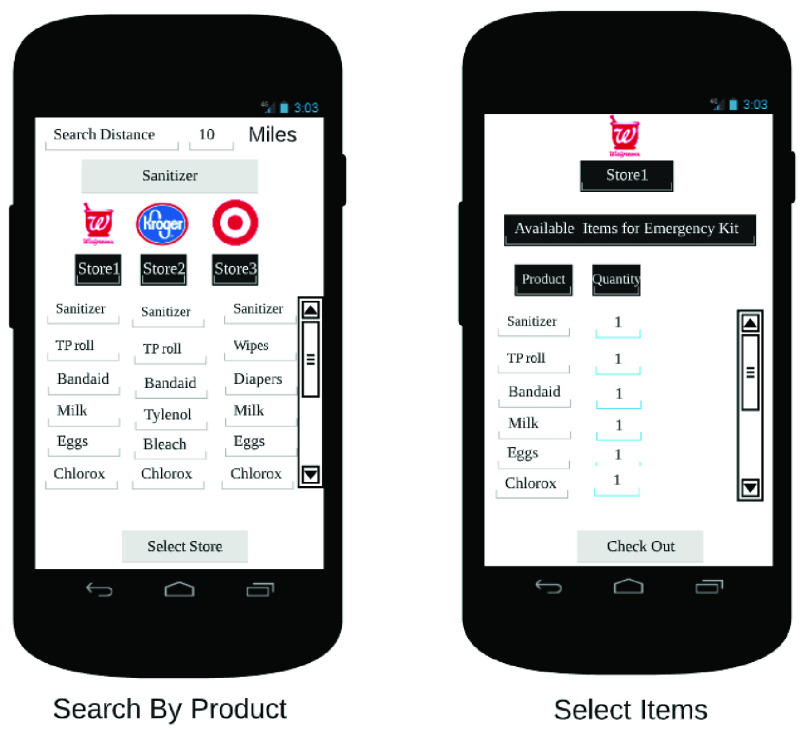
Inventory search.

**FIGURE 35.**
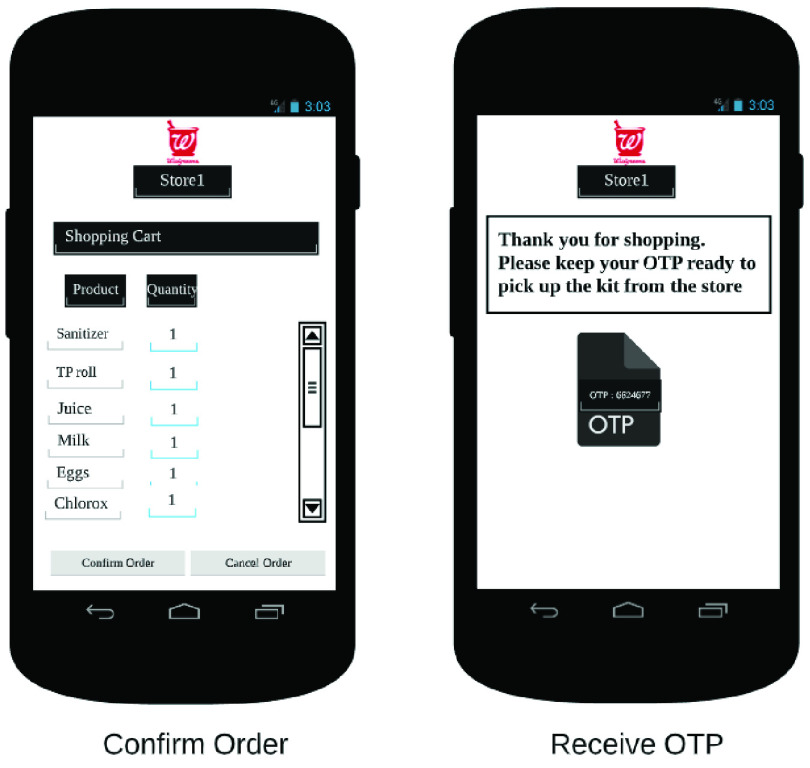
Place order.

**FIGURE 36.**
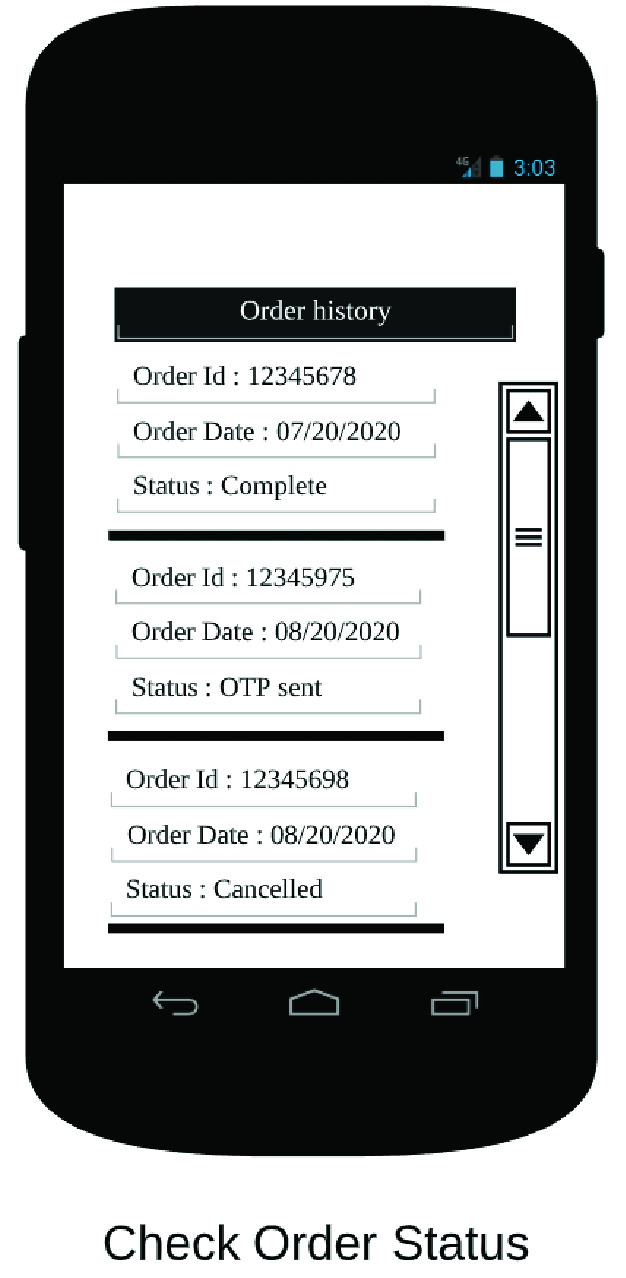
Check order status.

**FIGURE 37.**
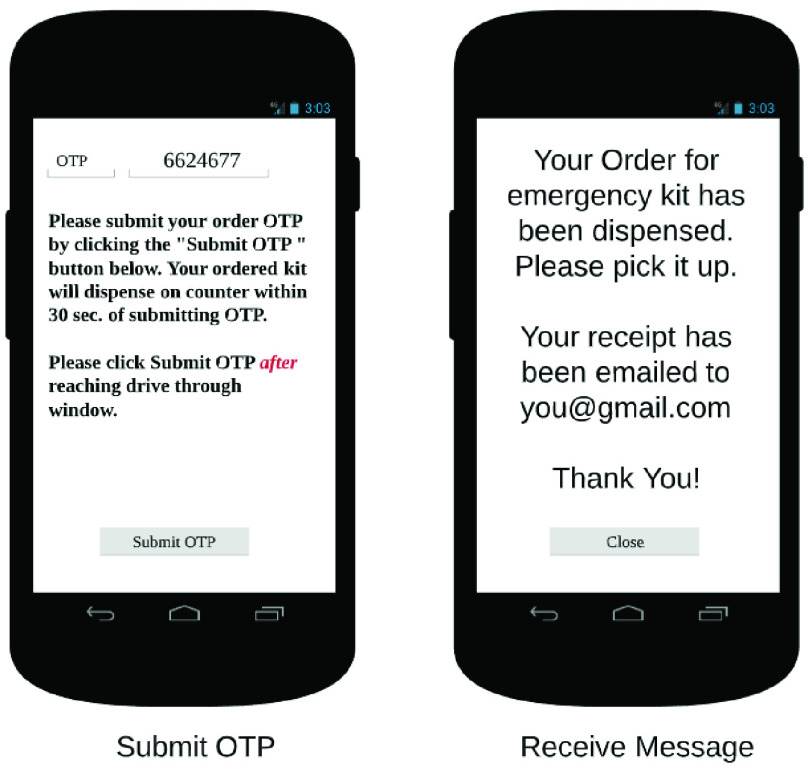
Pick kits.

**FIGURE 38.**
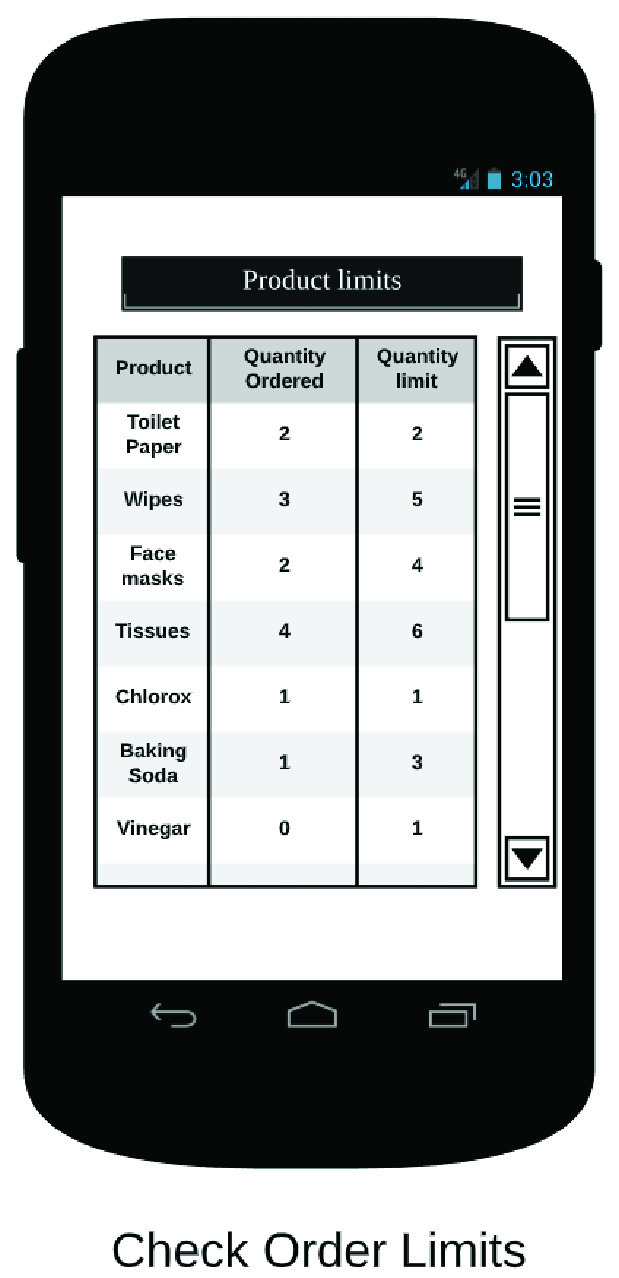
Check order limits.

The end user app is the primary interface between the RDS and the customer. It allows the customer to place orders on the RDS, in addition to other functionalities. In Figures 32–38 we present designs of some of the key functionalities of the end user app. As the design of the app illustrates, one of the primary design objectives was to keep the app simple, intuitive and easy to use.

### B. Order Management System Design

**FIGURE 39.**
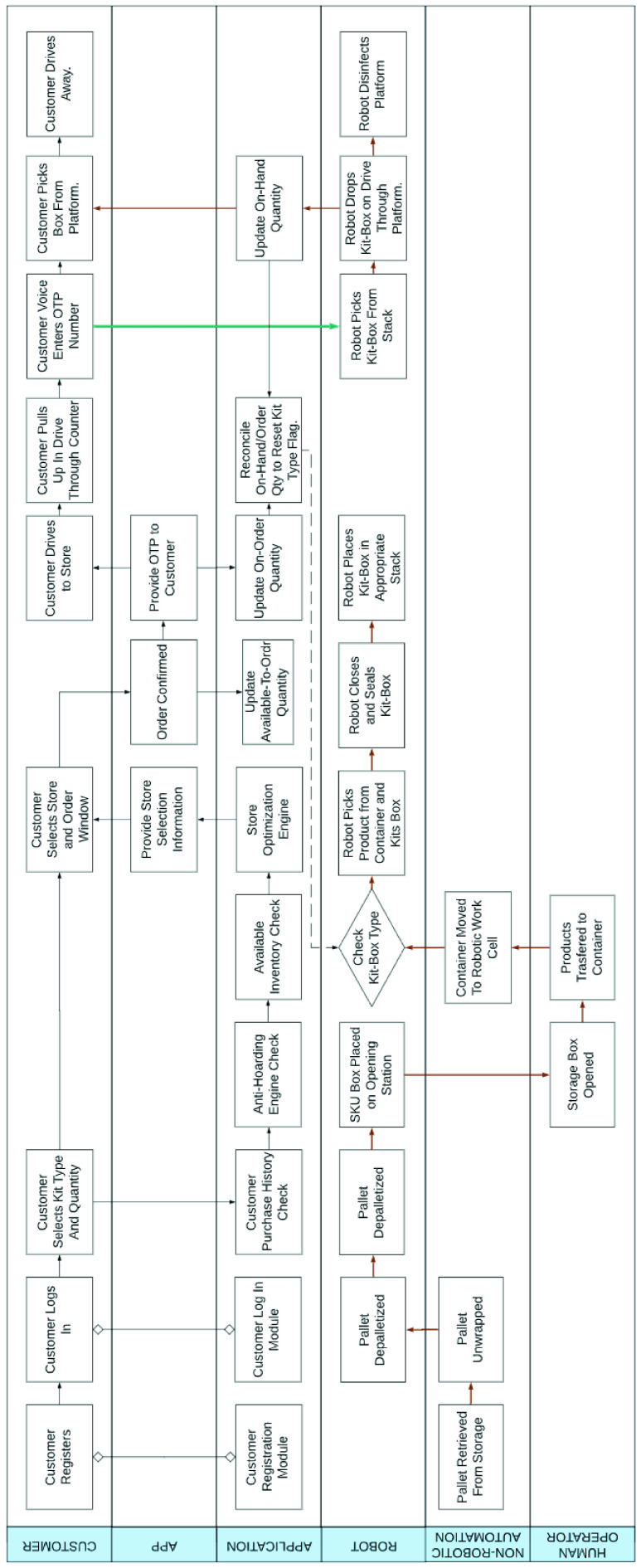
Automated system for distribution of survival Kit during pandemics.

**FIGURE 40.**
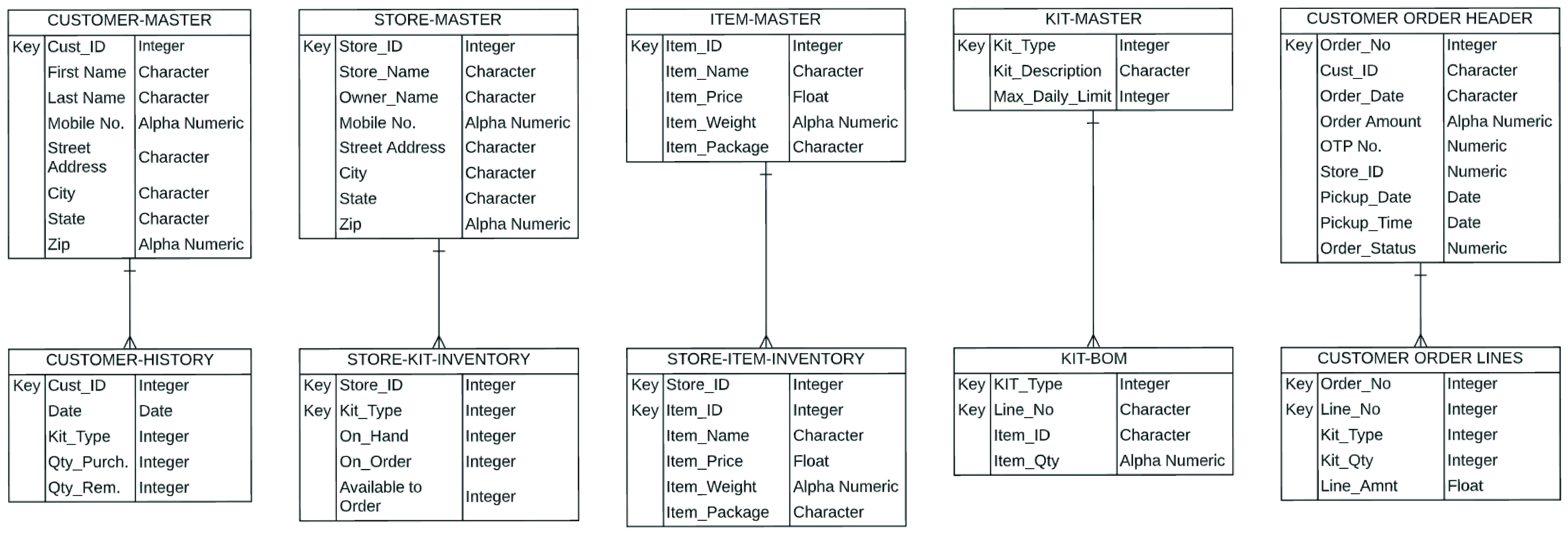
Database design for order management system.

While the end user app is focused on customer interaction, the order management system interfaces with the cyber layer of the RDS, in order to fulfill customer orders. In addition, the order management system contains the functionality for preventing hoarding, price-gouging and welfare fraud. In Figure 39, we present a *process design* of the order management system that helps achieve these desired functionalities. A *database design* for the order management system is presented in Figure 40.

## XIII. Cycle Time Analysis

While the design, simulation and validation of the RDS has established its technical feasibility, it does not confirm performance at the complete system level. Specifically, it does not answer key questions that an organization interested in deploying this system might have: (a) What is the rate at which orders can be filled by the proposed system, (b) Given a desired throughput rate, what is the most optimal work-cell configuration (c) Given a desired throughput rate, how much capacity investment is needed?, (d) What is the estimated customer wait time at the counter?

To arrive at these answers, we need to complete a throughput and cycle time analysis of the system. To determine the cycle times, the finalized work-cell design was run in the simulation software in a mode which collects cycle time data. Since the simulated work-cell is a digital twin, the generated cycle times represent what would be achieved by a real-world physical counterpart.

The cycle time for kitting one order is 32.34 seconds/box. Provisioning some time for a customer to pick up the order-box and drive away, we assume an order fulfillment rate of 1 order/minute. This implies that a robot with a 20 hour operation can serve 1200 orders in a day. Daily order volumes achieved for different shift hours and capacity investment levels is tabulated in Table 12.

**TABLE 11:** Stakeholder Needs to Component Features Mapping

	Information Systems Layer							Cyber-Physical Layer				Physical Layer											Full System			
	App		Order Mgt. System					Controller		Sensor		Robot			Gripper		Order Box		Tote		Dispenser		RDS System			
	Simple User trierface	Imentery Scarch	Order Reserve Freature	Queue Maragement Module	Anti Hoarding Module	Price Gouging Filter	Fraud Prevention Algorithm	Simple Proprantming Languap.	Powerful Language	Identify Grip Serface	Identify Object to Pick frem Pile	Fast	Low Cost	Small Footprint	Pick variety of items	Pick from Pile	Optimal size	Easy Sealing	Optimal Size	Low Cost	Contactless	Disinfoction	No Human in Leop	Simple Interfaces	Modular	Low Skill Operation
Customer Needs																										
Easy ordering	•																									
Inventory visibility		•	•																							
Quick Pick Up				•								•									•					
No Contagion																					•	•	•			
Store Needs																										
Low Cost								•		•	•		•		•	•		•	•	•	•		•			•
Reconfigurable									•	•	•				•	•					•			•	•	
Small footprint										•	•			•	•	•	•	•	•		•					
Easy to implement								•	•	•	•				•	•		•			•		•	•	•	•
Easy to operate								•		•	•				•	•	•	•	•		•		•	•	•	•
Societal Needs																										
No Hoarding					•																					
No Price Gouging No						•																				
Fraud							•

**TABLE 12:** Daily Order Volumes

	No. Of Work-cells Deployed				
Hours Per Day	1	2	3	4	5
8 hrs.	480	960	1440	1920	2400
16 hrs.	960	1920	2880	3840	4800
20 hrs.	1200	2400	3600	4800	6000
24 hrs.	1440	2880	4320	5760	7200

While it is theoretically possible to keep increasing the daily volume of orders served from a particular location, other constraints such as traffic congestion may come into play. Deciding on the volume of orders to be served from a location may need to be determined in the context of a much larger set of design parameters at the larger system level.

## XIV. Operational Life Analysis

An often cited advantage of robots is their ability to work non-stop. However, robots do have limits on their ability to sustain continuous operations. There are two primary reasons why robots may fail in the field. One is overheating of the robot motors and the other is mechanical failure of gears. These can be prevented by slowing down the motion, reducing the payload (which is usually not an option), or, adding some rest periods to break down continuous motion. It is akin to a weightlifter lifting a weight, and needing to rest to let his muscles recover, before resuming.

In pandemic situations, there are likely to be time periods of elevated demand when the RDS is required to run continuously for long periods of time. Hence, it is critical to have an idea of the robot’s ability to operate continuously, without failing. In the past, the only way to determine this was to run a physical prototype of the system, to its breaking point. However, the ROBOguide simulation software allows us to estimate the operational resilience of a robot for a given payload and operational constraints. In what follows, we present an analysis of the digital simulation data on duty cycle and gear life of the robot.

### A. Duty Cycle Analysis

**FIGURE 41.**
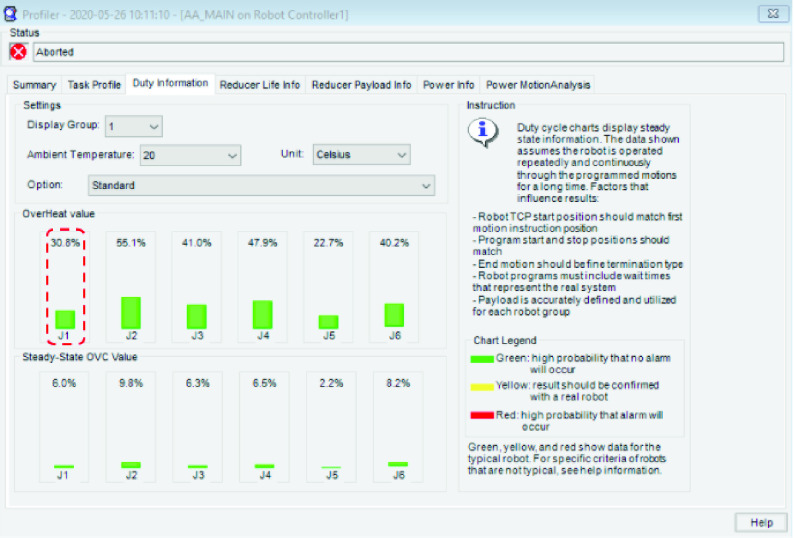
Robot duty cycle analysis.

In a robot, if more heat is generated for a given motion than can be dissipated by the motor, the motor will eventually overheat and fail after crossing a limit. This limit may vary across different robot models and is measured by the *duty cycle* of the robot. The duty cycle of a robot measures its ability to operate continuously at the specified speed and payload, without overheating of motors. Since we have set up a digital twin of the actual physical system, we are able to collect data on the duty cycle from the simulation run. The duty cycle data for the proposed set up is presented in Figure 41. The OverHeat value is a measure of the amount of heat generated by each axes’ motor as a result of the current input into it. For example, the J1-axis motor generates 30.8% of the max heat it can tolerate before faulting due to overheating. The green bars for each of the six axes of the robot imply that there is a high probability that none of the axes motors will get overheated during continuous operation.

### B. Gear Life Analysis

**FIGURE 42.**
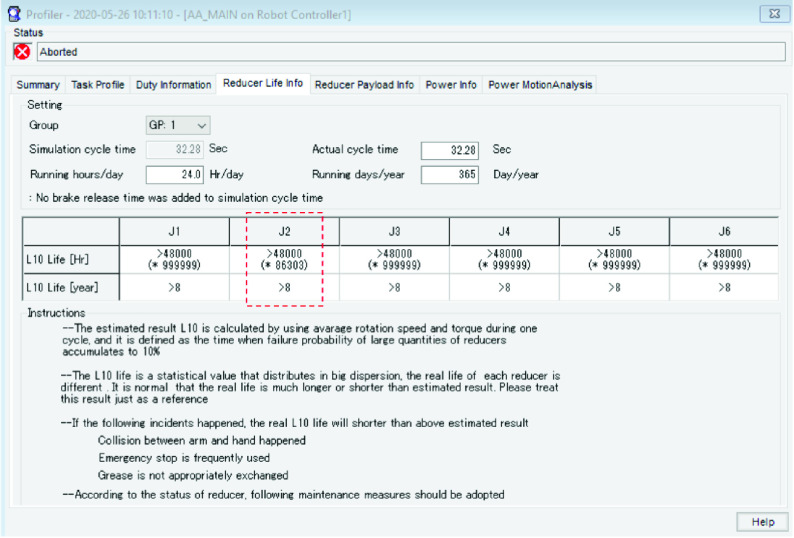
Robot gear life analysis.

Similar to the duty cycle data, the simulation run allows us to collect data on the expected gear life for each of the axes of the robot. Figure 42 lays out the expected gear life for each of the six axes of the robot. For example, the J2-axis gear is expected to last 86303 hours based on the motion profile and payload of the simulated cycle. This is more than the rated life of eight years (48000 hrs.) for the J2-axis gear.

In summary, based on the duty cycle and gear life analysis, we can say with a high degree of confidence that the robot can be operated $24\times 7$X365 for the entirety of its rated 8 year life without failing under the given operating conditions.

## XV. Stakeholder Requirements Mapping

We now look at the wider context in which the RDS is situated. The RDS is more than a complex engineered system. It is a socio-technical ’system of systems’, which, in order to be successful, must meet the needs of multiple stakeholders. The three main stakeholders of the RDS are the end customer, the store or organization implementing the RDS, and, society in general.

The needs of the customer are: reduced chances of contagion, availability of items, visibility into available inventory, large assortment of items, fair prices, easy and quick pick up, and, a simple and intuitive customer interface. The needs of the store are: low system cost, compact design, small footprint, ease of installation, ease of integration with existing store operations, ease of integration with existing store IT systems, ease of integration with third party delivery services, simple operation with low skill needs, easy reconfigurability, and, the ability to scale system throughput, depending on need. The societal needs are: fair and equitable distribution of essentials, and, minimizing the contagion of unproductive human behaviors like hoarding, price-gouging and welfare fraud.

These needs are satisfied by the different components making up the RDS. Because the RDS is a complex system with multiple components, the linkages between the individual stakeholder needs and system component features may become cognitively intractable. To clearly explicate these linkages, we present in Table 11, a mapping between the stakeholder needs and the functional features of the various components. The components are logically grouped into the information systems layer, the cyber-physical layer, the physical layer and the full RDS system.

A close look at the table reveals some key insights. While the physical layer prevents contagion, the information systems layer prevents the contagion of unproductive human behavior. Also, careful considerations at the overall system level, are needed for achieving a modular, reconfigurable and scalable system.

## XVI. Limitations

In this paper, we have proposed a comprehensive system, which has been designed and validated for real world implementation. However, some limitations remain.

First, this study is set in the context of a developed world economy and our design assumes vehicle ownership by the target population. In emerging market contexts where not everyone owns a vehicle, this configuration may not be applicable. However, the RDS as designed, is modular and a different dispense mechanism can be designed and integrated with a down-stream delivery mode which does not assume vehicle ownership.

Second, the proposed model may face congestion problems. Similar to foot traffic in stores, orders placed on the RDS may be lumpy in nature. This can lead to long queues during elevated demand time periods. This is likely to be similar to the lines of vehicles in drive-through windows of coffee shops during morning rush hour. Lessons learnt in managing queues from such operations may need to be incorporated to manage congestion.

Third, the current design limits the assortment size available to customers. Our current design assumes a single SKU item per tote. Hence, the number of items in the assortment is limited to the number of totes that can fit in the turntable. In the embodiment presented in this paper, we have seven slots available for the totes. We do not present multiple SKUs in the same tote because it is possible to have scenarios where only a single SKU is visible on the surface and the rest are buried deep inside. It may be possible to make changes in the design of the hardware as well as algorithms to allow multiple SKUs to be presented in a single tote.

Fourth, the system as designed, is limited to items that are not exposed or frozen. If we include items such as fruits which come in direct contact with the robot gripper, we will need to use food grade robots, approved by the FDA. Food grade robots are significantly more costly than regular robots. This will increase the cost of the system by orders of magnitude, which is likely to get in the way of widespread deployment needed to serve those in most need. Hence, we are limited to items that are sealed and do not come in direct contact with the robot gripper. Similarly, our assortment does not include frozen items because that would require additional equipment set up in the work-cell, further increasing the cost of the system. With additional captial investments it is possible to lift these constraints.

Fifth, although remote, there remains a possibility for germ transmission. The current design requires the customer to open her window to collect the order-box, from the pick-up platform, exposing her to a limited extent. It may be possible to design dispense mechanisms which do not require the customer to open her vehicle window. An enhancement like this should completely eliminate the risk of contagion from the RDS system.

Sixth, there remain limits to the volume of orders the RDS can complete in a day. In our throughput analysis, we found that a single system, operating round the clock, can serve 1440 orders per day. While this volume may be sufficient in most cases, larger communities might need a higher throughput rate. The order volume can be increased by deploying multiple work-cells, but, any expansion of capacity needs to be planned in light of wider system level considerations such as traffic patterns and congestion management.

Finally, in the RDS system as designed, there is a customer wait time of close to a minute. It might be possible to reduce, or, completely eliminate this wait time by varying the configuration and designs of the cyber, physical, cyber-physical and information system layers.

## XVII. Conclusion

One of the most fundamental responsibilities of a society is to ensure that all people have access to enough food and essential medical supplies. Though it may not be easily visible, the COVID-19 pandemic seems to have created parallel universes in the last mile delivery of food and essential supplies. One of these runs on the digital highway and enables fast and convenient delivery with minimal or no exposure to germs. In this universe, people with sufficient means are able to divert the flow of goods to their doorsteps, for a premium, from the safety of their homes. In the other universe, consumers have to search through multiple stores for scarce supplies. This considerably increases the risk of exposure in an essential activity that every household must engage in. The lack of equity in safe access to food and essentials might partly explain the disproportionately high mortality rate among the poor during pandemics, as revealed in CDC data on COVID-19 deaths [8].

Our research is motivated by a desire to minimize this *poverty-penalty* in access to essentials during pandemics. We have proposed a robotic drive through system which can be deployed to achieve this objective. We have designed, simulated and validated the system to ensure readiness for implementation in the field. To ensure ease of deployment, the system has been designed using widely available components. Open source designs of artefacts, developed as part of this study, are available upon request, to organizations interested in serving their communities.

## Figures and Tables

**FIGURE 43.**
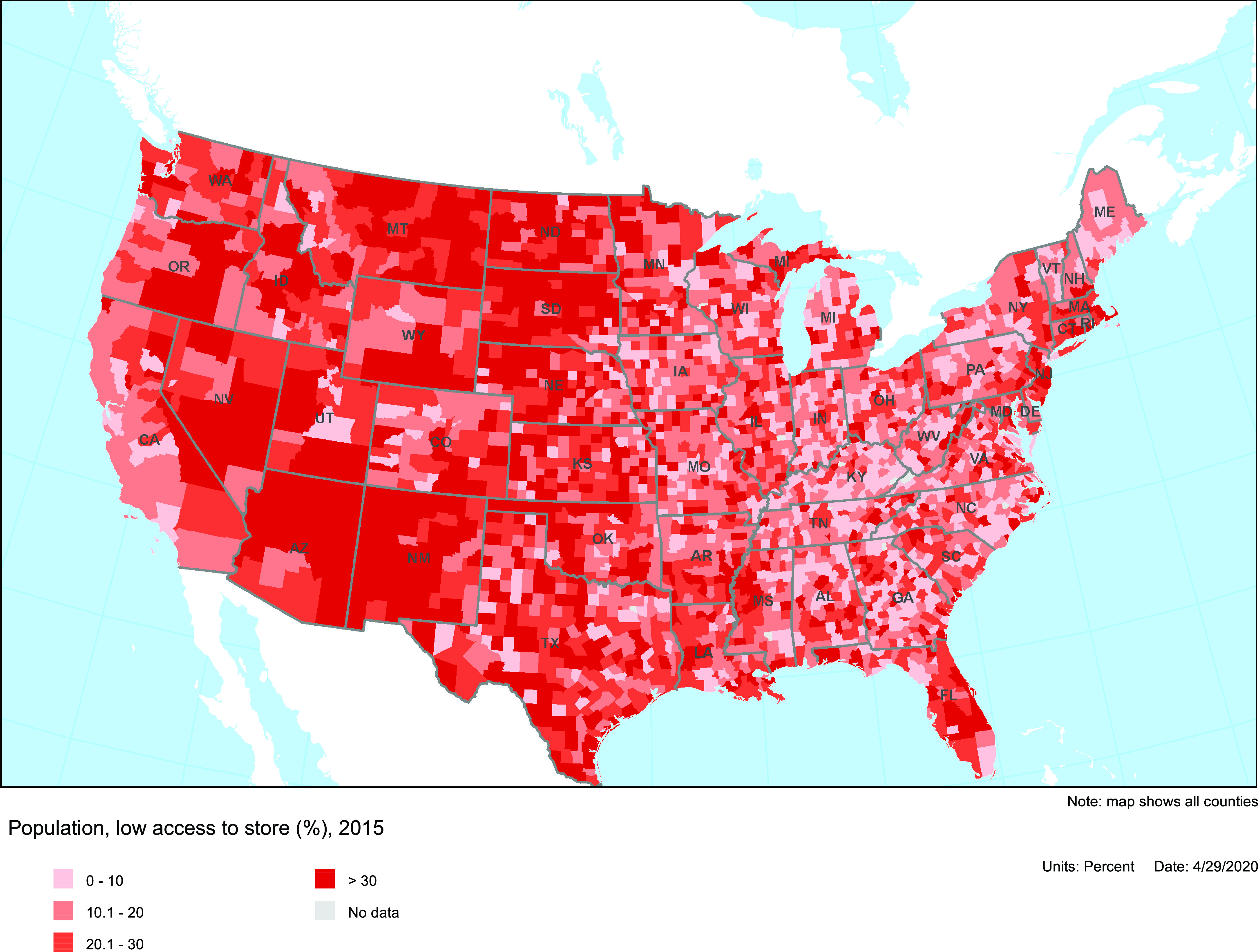
Access to stores in America (Source: USDA Economic Research Service, ESRI.)

## Appendix A Access to Stores in America (Source: USDA)

See Figure 43.
